# Immunoglobulin gene rearrangement *IGHV3-48* is a predictive marker of histological transformation into aggressive lymphoma in follicular lymphomas

**DOI:** 10.1038/s41408-019-0213-9

**Published:** 2019-06-17

**Authors:** María García-Álvarez, Sara Alonso-Álvarez, Isabel Prieto-Conde, Cristina Jiménez, M. Eugenia Sarasquete, M. Carmen Chillón, Alejandro Medina, Ana Balanzategui, Rebeca Maldonado, Alicia Antón, Noemí Puig, Marta Rodríguez, Oscar Blanco, Pilar Tamayo, Verónica González-Calle, Alejandro Martín, Ramón García-Sanz, Marcos González, M. Dolores Caballero, Miguel Alcoceba

**Affiliations:** 1 Department of Hematology, University Hospital of Salamanca (HUS/IBSAL), Salamanca, Spain; 2 Department of Hematology, Central University Hospital of Asturias (HUCA), Oviedo, Spain; 3 Biomedical Research Networking Centre – Oncology (CIBERONC), Madrid, Spain; 4 Department of Pathology, University Hospital of Salamanca (HUS/IBSAL), Salamanca, Spain; 5 Department of Nuclear Medicine, University Hospital of Salamanca (HUS/IBSAL), Salamanca, Spain; 6 Cancer Research Centre - IBMCC (USAL-CSIC), Salamanca, Spain

**Keywords:** B-cell lymphoma, Risk factors

## Abstract

Follicular lymphoma (FL) is a heterogeneous disease whose pathogenesis remains partially unknown. Around 20% of FL patients experience early progression or treatment-refractory disease and 2–3% of patients per year experience histological transformation (HT) into a more aggressive lymphoma (tFL). Here, we evaluate the immunoglobulin heavy chain variable (*IGHV*) gene usage and mutational status in 187 FL cases to assess its impact on clinical outcome and histological transformation. The *IGHV* gene repertoire was remarkably biased in FL. The *IGHV4-34* (14%), *IGHV3-23* (14%), *IGHV3-48* (10%), *IGHV3-30* (9%) and *IGHV3-21* (7%) genes accounted for more than half of the whole cohort. *IGHV3-48* was overrepresented in cases of tFL (19%) compared with non-transformed FL at 5 years (5%, *P* = 0.05). Patients with the *IGHV3-48* gene were significantly more likely to have had HT after 10 years than those who used other genes (71% vs. 25%, *P* < 0.05), irrespective of the therapy they received. Moreover, *IGHV3-30* was also overrepresented in cases of FL (9%) and tFL (13%) compared with diffuse large B-cell lymphoma in which it was nearly absent. In conclusion, our results indicate a role for antigen selection in the development of FL, while the use of *IGHV3-48* could help predict histological transformation.

## Introduction

Follicular lymphoma (FL) is the most common indolent non-Hodgkin lymphoma (NHL, 20–30%), with an incidence of 12.5 new cases per 100 000 inhabitants/year. FL is typically characterized by a non-aggressive nature, usually responds successfully to first-line therapy, and has a median survival of 15 to 20 years^1–3^.

FL is considered an incurable disease that is characterized by a pattern of multiple relapses, a decreasing duration of response, and a gradual acquisition of resistance to different drugs. Despite the improvement in treatment effectiveness due to the incorporation of immunochemotherapy regimens, including anti-CD20 monoclonal antibody, 20% of patients experience early progression or develop a treatment-resistant disease within 2 years of receiving first-line therapy^2,4,5^. Furthermore, FL can undergo histological transformation (HT) into a more aggressive lymphoma, most commonly to diffuse large B-cell lymphoma (DLBCL), with a transformation rate of 2–3% per year^6–8^. Transformed follicular lymphoma (tFL) involves a clonal relationship between the initial FL and the aggressive form and it is considered to be one of the most unfavorable events in the natural history of FL^9^, because transformation has customarily been associated with low cure rates with conventional therapies and short survival^10^. Several prognostic indices for FL, such as the Follicular Lymphoma International Prognostic Index (FLIPI)^11^, FLIPI2^12^ or the recently described complete response rate at 30 months (CR30)^4^ have been defined because of the need to identify high-risk group of patients. However, FLIPI perform poorly in terms of specificity to predict transformation^13^, and the remaining indexes are not useful to predict histological transformation. Unraveling the biology and pathogenesis of this disease would improve the identification of poor-prognosis groups at the time of diagnosis and might enable risk-adapted treatment strategies to be employed.

FL arises from the clonal proliferation of germinal center (GC) B-cells, which have experienced malignant transformation, resulting in a follicular pattern with architectural features typical of normal germinal centers^3,14^. These GC B-cells harbor a clonal rearrangement of the immunoglobulin heavy chain gene (*IGH*), which occurs during B-cell differentiation to generate a completely functional variable (*IGHV*)-diversity (*IGHD*)-joining (*IGHJ*) junction in the *IGH* gene. The *IGHV* gene repertoire and somatic hypermutation status has been extensively analyzed in B-cell lymphomas, showing a biased use of *IGHV* genes in chronic lymphocytic leukemia (CLL), DLBCL, splenic marginal zone lymphoma (SMZL), Waldenström macroglobulinemia (WM), mantle cell lymphoma (MCL) and primary central nervous system lymphoma^15–22^. Some *IGHV* genes have also been associated with clinical outcome. Thus, the unmutated status of the *IGHV* rearrangement, or the presence of those *IGHV3-21* belonging to the stereotyped B-cell receptor subset #2 are associated with unfavorable prognosis in CLL^16,17,23,24^.

In the case of FL, the *IGHV* repertoire has only occasionally been reported in small series, and without any clinical associations^25–27^. Recently, Berget et al. reported an association in a series of 106 FL patients between the presence of *IGHV5* or to use more than one *IGHV* subgroup and poor survival^14^. However, there is no information about the role of *IGHV* rearrangements in the treatment response or the risk of HT.

The aim of the present study was to analyze in detail the use of *IGHV*, *IGHD* and *IGHJ* genes and the somatic hypermutation (SHM) rate in the largest FL series to date, to improve our understanding of the biology of FL and its impact on clinical outcome and the risk of histological transformation.

## Patients and methods

### Patient characteristics

The study included a total of 187 consecutive patients with histologically confirmed FL grade I-IIIA according to the 2017 WHO classification^3^ diagnosed between January 1995 and May 2017 (Supplemental Fig. S1). FL grade IIIB and composite cases of FL+DLBCL were excluded^28^. Cases without biological sample and/or clinical data were also excluded.

Clinical characteristics of the cohort are described in Table 1. The median age at diagnosis was 58 years (range 19–87 years) and 53% of the patients were females. Within our series, 36% of the patients were high-risk according to FLIPI criteria. One hundred sixty-three patients received treatment, of whom 98 (60%) received rituximab-based immunochemotherapy (R-ICT) -R-CHOP therapy in most of them (68/98 cases, 69%)-, and 65 (40%) received other schemes without rituximab. Seventy-three patients (39%) also received rituximab as maintenance. The other 24 patients (13%) never were treated until the time of last follow-up or HT into aggressive lymphoma.

**Table 1:** Clinical characteristics of FL patients (*n* = 187)

Variable	Training cohort n (%)
Age, years (median, range)	58 (19–87)
Sex F/M	99 (53/8847)
Histological grade^a^	
I	84 (48.8)
II	67 (39)
IIIA	21 (12.2)
FLIPI^a^	
0–1 (Low risk)	49 (32.9)
2 (Intermediate risk)	46 (30.9)
3–5 (High risk)	54 (36.2)
Ann Arbor^a^	
I	24 (14.2)
II	18 (10.7)
III	18 (10.7)
IV	109 (64.5)
First-line therapy	
Never treated	24 (12.8)
Rituximab-based ICT	98 (52.4)
R-CHOP	68 (69.4)
R-Bendamustine	11 (11.2)
R-CVP	3 (3.1)
Others	16 (16.3)
CT without rituximab	48 (25.7)
CHOP	35 (72.9)
Fludarabine-based	3 (6.3)
Others	10 (20.8)
Radiotherapy alone or with rituximab	13 (7)
Rituximab alone	4 (2.1)
Maintenance with rituximab^a^	73 (39)
Response after induction therapy	
CR	87 (53.7)
PR	63 (38.9)
NR/Failure	12 (7.4)

*ICT* immunochemotherapy, *R* rituximab, *CHOP* cyclophosphamide, doxorubicin, vincristine, prednisone, *CVP* cyclophosphamide, vincristine, prednisone, *CT* chemotherapy; *CR* complete response, *PR* partial response, *NR* no response, *FLIPI* FL International Prognosis Index

^a^Histological grade was available for 172 (92%) patients; FLIPI was available for 149 (80%) patients; Ann Arbor was available for 169 (90%) patients; Maintenance was calculated for 147 (79%) patients

This study was approved by the Ethical Committee of the University Hospital of Salamanca in accordance with Spanish law and the Declaration of Helsinki. Written informed consent was obtained from all participants.

### DNA extraction

DNA was isolated on samples at the time of diagnosis of FL, and in the biopsy of those cases with documented HT. gDNA from fresh-frozen tissue (65% of cases) was isolated by the phenol-chloroform method^29^ or the Maxwell® 16 System (Promega, Madison, WI, USA). In cases in which DNA was extracted from bone marrow (20%), peripheral blood (2%) and other tissues (3%), the Maxwell® 16 System (Promega) or DNAzol reagent (MRC, Cincinnati, OH, USA) was used^30^. Finally, gDNA extraction from formalin-fixed paraffin-embedded (FFPE) tissue (10%) was isolated using the RecoverAll Total Nucleic Acid Isolation Kit (Ambion/Applied Biosystems, Foster City, CA, USA) or the QIAamp DNA FFPE Tissue Kit (Qiagen, Hilden, Germany).

DNA was quantified in a NanoDrop 1000^TM^ Spectrophotometer (Thermo Fisher Scientific, Waltham, MA, USA). The quality and purity of the gDNA extracted from FFPE tissue, were assessed with the 4200 TapeStation (Agilent Technologies, Santa Clara, CA, USA) system, using the Genomic DNA ScreenTape assay.

### *IGH* rearrangements amplification

*IGH* rearrangements were amplified according to the BIOMED-2 Concerted Action protocols^31^, in which the complete V-D-J rearrangement was amplified by multiplex PCR with a set of six family-specific *IGHV* primers of the framework region 1 (FR1), and one *IGHJ* consensus primer. For the samples with no detectable amplification from FR1, PCR was performed from Leader or FR2 region. The presence of the monoclonal rearrangement was then confirmed by GeneScan with an ABI 3500xL DNA Sequencer (Applied Biosystems, Foster City, CA, USA).

### *IGH* rearrangement sequencing and identification

PCR products were sequenced in forward and reverse reads, using the same primers as for PCR amplification and the Big-Dye® Terminator v1.1 Cycle Sequencing Kit (Applied Biosystems)^31^. Sequencing was carried out with an ABI 3500xL DNA Sequencer (Applied Biosystems).

Complete V-D-J rearrangements and the percentage of germline identity were identified using the IMGT/V-QUEST software (http://www.imgt.org). Sequences with germline identity less than or equal to 98% were considered to be mutated, while those with >98% identity were considered to be sequences without somatic hypermutation (SHM) or to be unmutated.

### t(14;18) Nested PCR

The t(14;18) was amplified by nested PCR adapting the method of Gribben et al*.*^32^ using two BCL2 primers directed at major breakpoint region (MBR) and minor cluster region (mcr) in chromosome 18 and a consensus primer directed to J_H_ in chromosome 14. The J_H_ internal primer was marked with a fluorochrome for detecting BCL2 translocation size by GeneScan with an ABI 3500xL DNA Sequencer (Applied Biosystems).

### Definitions and statistical analysis

We explored the following clinical endpoints: (i) response to induction treatment according to International Working Group criteria, based on computed tomography (CT) scans (*n* = 130) or a ^18^-fluorodeoxyglucose-positron emission tomography (PET)/CT scan (*n* = 16);^33–35^ (ii) complete response rate at 30 months (CR30), defined as complete response 30 months after the date induction treatment began;^36^ (iii) failure-free survival (FFS), defined as less than a partial response at the end of induction, relapse, progression or death;^37^ (iv) time to transformation (TTT), measured as the time from the date of FL diagnosis to that of histologically confirmed transformation into aggressive lymphoma (tFL)^38^, defined solely on the basis of pathological criteria, getting biopsies at the time of FL diagnosis and when there was a clinical suspicion of tFL; (v) histological transformation at 10 years (10y-HT), defined as the probability that HT occurred at any time within a given 10-year period; and (vi) overall survival (OS), which is the time from the date of diagnosis of FL to that of death from any cause.

Possible associations between variables were analyzed by the χ^2^-square test for categorical variables, and by Student’s unpaired-samples t-test or the Mann-Whitney U test for continuous variables. P values were Bonferroni corrected (Pc) to take into account multiple testing. Survival was estimated by the Kaplan–Meier method and the differences were assessed by the log-rank test. Subsequently, all variables for which there was some indication of a significant association with clinical features in the univariate test (*P* < 0.1) were considered in a multivariate Cox regression model. Differences were considered to be statistically significant for values of *P* < 0.05. All analyses were performed using SPSS (IBM SPSS 23.0, IBM Corp, Armonk, NY, USA).

## Results

### *IGHV*, *IGHD* and *IGHJ* Gene Repertoire in FL

A total of 138 clonal V–D–J rearrangements out of 187 cases (74%) were detected in our study cohort. No clonal rearrangements were identified in the other 49 cases due to their highly polyclonal background (82%) or the absence of amplification arising from the small quantity or poor quality of the available DNA (18%). Most sequences were obtained from FR1 (80%). Clinical characteristics of the cohort with clonal *IGH* rearrangements (*n* = 138) in comparison with the global cohort are described in Supplemental Table S1.

**Fig. 1:**
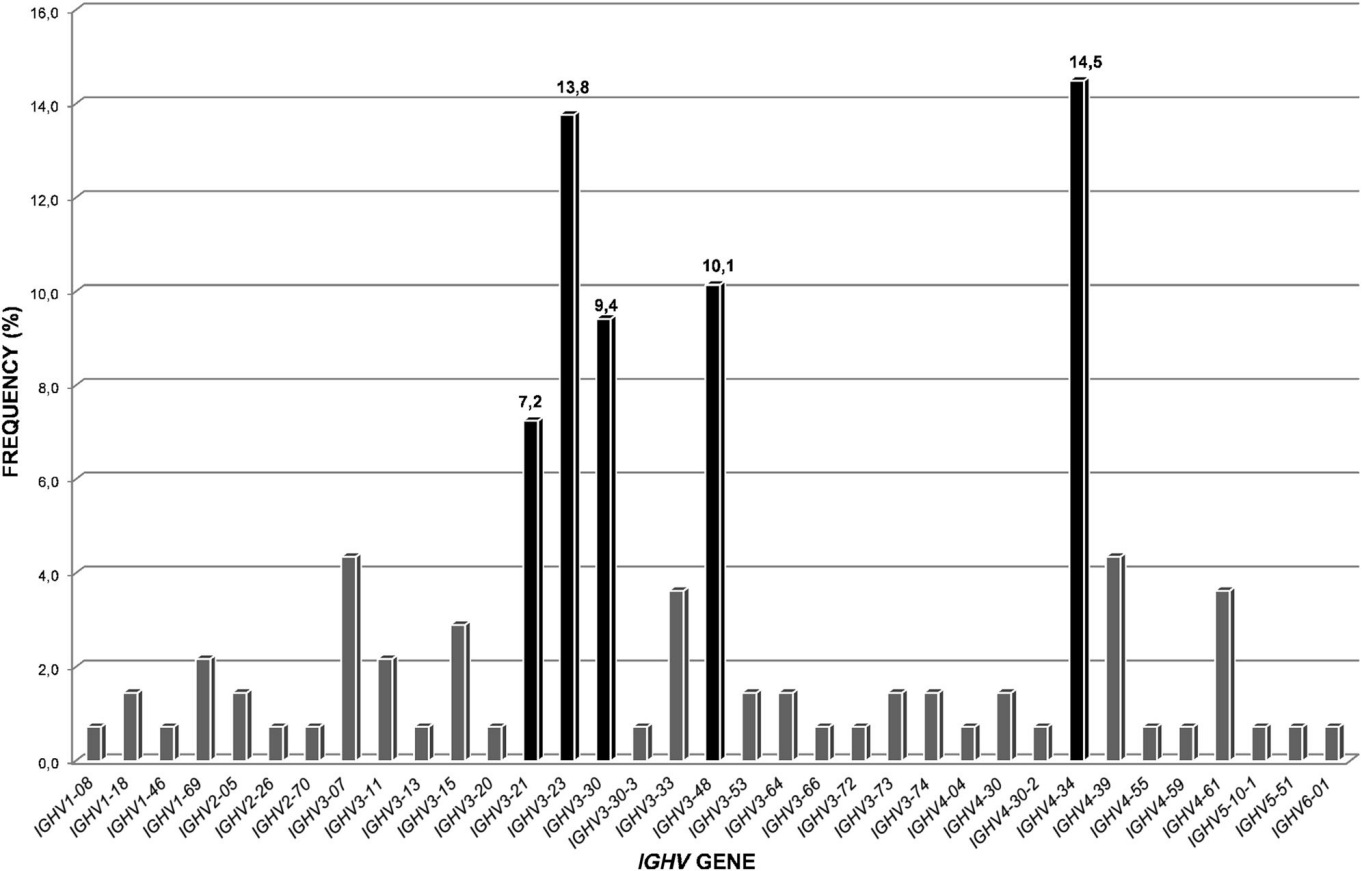
*IGHV* gene repertoire in follicular lymphoma (*n* = 138). The 35 *IGHV* genes expressed in our series are presented along the X-axis

*IGHV3* (63%) and *IGHV4* (27%) were the most frequent subgroups of clonal V-D-J rearrangements (Supplemental Table S2). A total of 35 functional *IGHV* genes were identified, being *IGHV4-34* (14%), *IGHV3-23* (14%), *IGHV3-48* (10%), *IGHV3-30* (9%) and *IGHV3-21* (7%) the most represented (Fig. 1 and Supplemental Table S3). Of the *IGHD* genes, *IGHD3* (37%) and *IGHD2* (25%) were the most frequent subgroups (Supplemental Table S2), with *IGHD3-10* (12%) and *IGHD3-22* (10%) being the most frequently expressed (Supplemental Table S4). Finally, of the *IGHJ* genes, *IGHJ4* (48%) was the most prevalent gene in this FL series (Supplemental Table S2). Summary list of the *IGHV*, *IGHD* and *IGHJ* subgroups and gene usage in the present series are provided in Supplemental Tables S2–S4.

### Analysis of *IGHV* mutational status in FL and VH CDR3 length

Somatic hypermutation (SHM) was feasibly studied in 130 of 138 (94%) clonal V–D–J rearrangements. SHM was detected in 126 (97%) of them. The percentage of germline identity to the closest *IGHV* gene ranged from 67.2 to 100%, with a mean of 87.2%. No differences were observed in the proportions of patients with SHM between the *IGHV* subgroups (Supplemental Fig. S2).

Only four patients (3%) in our series were unmutated, three of them with a 100% germline identity and 99.8% in the other patient. Unmutated sequences were observed in the *IGHV1* and *IGHV3* subgroups (Supplemental Fig. S2); the affected genes were *IGHV1-69*, *IGHV3-21* and *IGHV3-30-3*.

The VH CDR3 region was identified in 113/138 (82%) cases with clonal V–D–J rearrangement. The median VH CDR3 length was 16 amino acids (range 8 to 28). There were no statistically significant differences in mean length between the subgroups or genes (Supplemental Table S5).

### Comparative Analysis of *IGH* Rearrangements in FL with Other B-LPDs and Normal B Cells

In order to identify unique characteristics of FL, we compared our FL dataset with the results reported for CD5-/IgM+normal B cells and other B-cell lymphoproliferative disorders (B-LPDs), namely, de novo DLBCL, MCL, CLL, SMZL, hairy cell leukemia (HCL), multiple myeloma (MM) and WM (Table 2 and Supplemental Table S6)^19,21,39–44^. The *IGHV1* subgroup was significantly underrepresented in FL relative to all B-LPDs (5% vs. 15 to 30%, *P* < 0.05, *Pc* < 0.05) except WM (7%). Conversely, *IGHV3* subgroup was overrepresented in FL relative to DLBCL (63% vs. 44%, *P* < 0.01, *Pc* < 0.05), CLL (48%, *P* < 0.01, *Pc* < 0.05), MCL (52%, *P* < 0.05, *Pc* < 0.1), and MM (49%, *P* < 0.01, *Pc* < 0.1), although statistical differences were lost after Bonferroni correction in the two latter cases.

**Table 2:** Comparison of the *IGHV*, *IGHD*, and *IGHJ* subgroups and significantly different *IGHV* in our series of FL with those reported for B-LPDs and CD5-/IgM+normal B cells

*IGH*	FL (*n* = 138)	DLBCL (*n* = 103)	MCL (*n* = 807)	CLL (*n* = 7596)	SMZL (*n* = 133)	HCL (*n* = 102)	MM (*n* = 270)	WM (*n* = 58)	CD5-/IgM+(*n* = 206)
*IGHV*									
1	5.1	**16.5** ^**^	**15.5** ^**^	**23.8** ^**^	**30.1** ^**^	**14.7** ^*^	**15.6** ^**^	6.9	**13.1** ^*^
2	2.9	1.9	1.5	3.4	0.8	2.9	6.3	0	1.9
3	63.0	**43.7** ^**^	51.6*	**48.2** ^**^	**49.6** ^*^	53.9	**48.9** ^**^	75.9	53.9
4	26.8	33.0	25.8	20.6	17.3	23.5	20.4	13.8	24.8
5	1.4	3.9	5.1	2.5	2.3	1.0	7.8	1.7	2.9
6	0	1.0	0.6	1.2	0	2.9	1.1	1.7	2.4
7	0	0	0	0.4	0	1.0	0	0	1.0
3–21	7.2	4.8	**16.5** ^**^	4.7	3.0	3.1	**1.5** ^**^	5.2	**0** ^**^
3–23	13.8	9.7	**7.4** ^*^	**8.5** ^*^	18.0	18.1	**5.0** ^**^	**29.3** ^*^	12.1
3–30	9.4	**0** ^**†**^	**3.5** ^**^	5.5	6.0	8.5	10.0	8.6	5.8
3–48	10.1	5.8	**3.6** ^**^	**3.9** ^**^	3.8	5.3	**1.8** ^**^	**0** ^*^	**4.4** ^*^
4–34	14.5	15.5	14.6	**8.9** ^*^	7.5	7.4	**0.9** ^**^	**0** ^**^	**3.9** ^**^
*IGHD*									
1	9.6	10.7	10.6	8.2	5.2	12.7	7.6	27.3	6.4
2	24.8	21.4	17.4	19.6	15.5	12.7	24.2	4.5	21.2
3	36.8	35.9	34.2	40.3	46.6	41.2	25.8	18.2	37.2
4	12.0	5.8	8.5	6.3	7.8	6.9	10.6	13.6	10.9
5	4.0	9.7	8.3	8.8	9.5	6.9	13.6	9.1	10.3
6	12.8	14.6	20.3	15.9	15.5	18.6	12.1	27.3	10.3
7	0	1.9	0.6	0.7	0	1.0	6.1	0	3.8
*IGHJ*									
1	0.8	3.9	0.6	1.8	1.5	2.9	1.4	4.4	1.0
2	2.4	4.8	3.5	2.3	0	3.9	1.4	8.9	2.0
3	11.9	12.6	7.6	9.9	14.3	9.8	17.8	13.3	8.0
4	47.6	44.7	43.7	13.3	37.6	46.1	53.4	35.6	55.0
5	17.5	12.6	15.0	10.5	19.5	12.7	8.2	13.3	10.0
6	19.8	21.4	29.6	32.3	27.1	24.5	17.8	24.4	24.0

Frequencies expressed as percentages. References for comparisons are as follows: DLBCL^21^, MCL^40^, CLL^41^, SMZL^42^, HCL^43^, MM^44^, WM^19^, and normal B cells^39^. Significant differences between FL and other B-LPDs or normal B cells are depicted in bold

*MCL* mantle cell lymphoma, *CLL* chronic lymphocytic leukemia, *SMZL* splenic marginal zone lymphoma, *HCL* hairy cell leukemia, *MM* multiple myeloma, *WM* Waldenström macroglobulinemia

^*^P < 0.05, ^**^P < 0.01

*IGHV3-21* (7%), *IGHV* genes that are frequently used in FL, was completely absent from CD5-/IgM+normal B cells (*P* < 0.01, *Pc* < 0.05) and relatively underrepresented in other B-LPDs^19,21,39–44^. There were significant differences in the frequency of the *IGHV3-30* gene, which was absent from DLBCL (*P* < 0.01, *Pc* < 0.05)^21^. *IGHV4-34* was more frequent than in FL (14%) than in CD5-/IgM+normal B cells (4%, *P* < 0.01, *Pc* < 0.05), MM (1%, *P* < 0.01, *Pc* < 0.05) or WM (0%, *P* < 0.01, *Pc* = 0.06)^19,39,44^.

### Comparative Analysis of *IGH* Rearrangements in tFL with FL and DLBCL

In the FL series, 47 patients (25%) underwent HT into aggressive lymphoma (tFL). The clonal relationship between the paired samples (FL/tFL) was confirmed in 45/47 cases (96%) by BIOMED-2 or BCL2-*IGH* breakpoint analysis. Two patients were excluded because they expressed different clones at diagnosis and transformation. A total of 31 clonal V-D-J rearrangements out of 45 tFL cases (69%) were detected (31/138 (22%) total cases with *IGHV* gene available).

With the purpose of identifying singular characteristics of tFL, we compared these cases with those of FL without HT for a minimum of 5 years follow-up (*n* = 63), and germinal (GCB, *n* = 32) and non-germinal (non-GCB, *n* = 48) center B-cell-like de novo DLBCL previously analyzed by our group^21^. The *IGHV1* subgroup was significantly underrepresented in tFL relative to the non-GCB DLBCL (3% vs. 23%, *P* < 0.05) while the *IGHV3* subgroup was overrepresented in tFL compared with non-GCB DLBCL cases (74% vs. 40%, *P* < 0.01) (Supplemental Tables S7–S10).

**Fig. 2:**
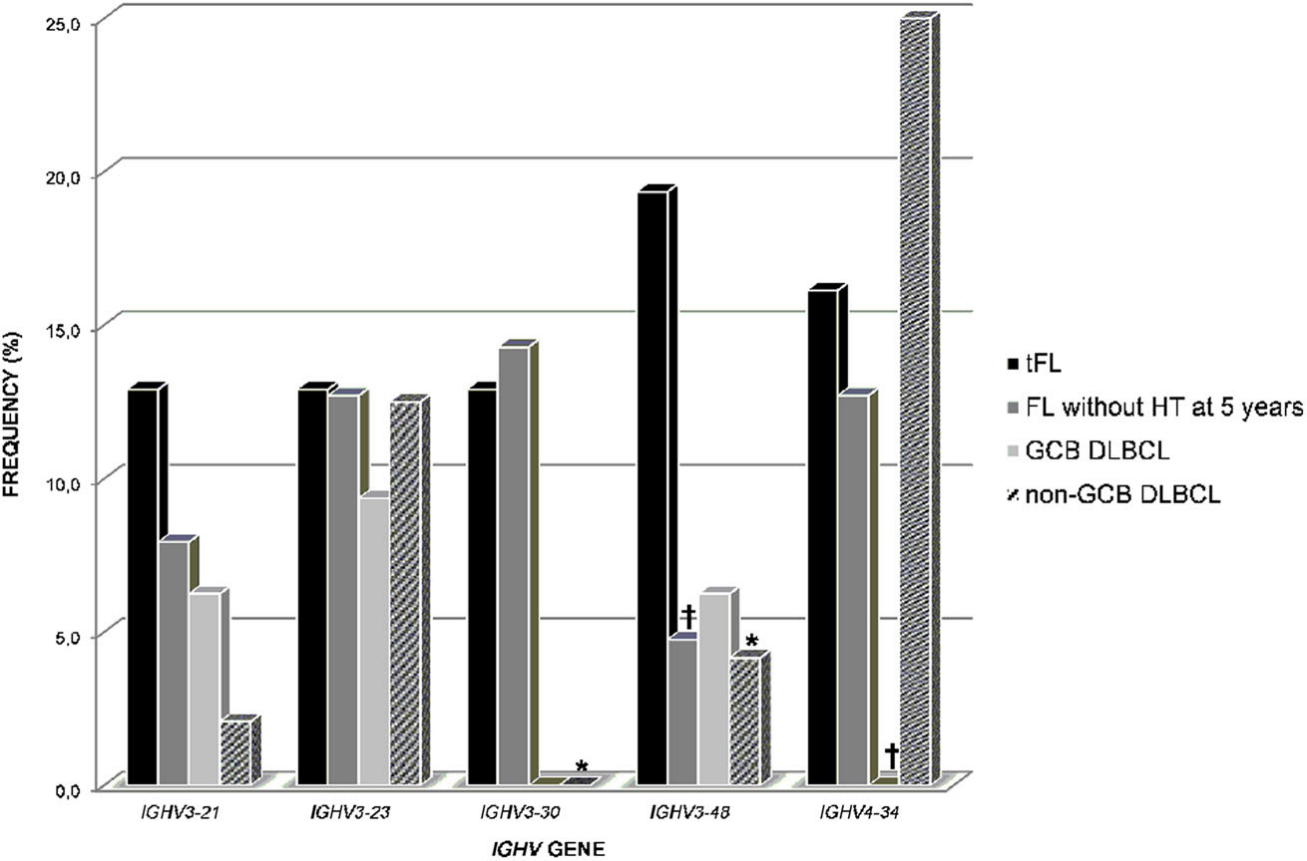
Comparison of the *IGHV* gene usage in tFL with FL without HT at 5 years and GCB/non-GCB DLBCL. The five *IGHV* genes most frequently expressed in our series are shown along the X-axis. *P < 0,05. GCB, germinal center B-cell-like; non-GCB, non-germinal center B-cell-like

The *IGHV3-48* gene was more frequent in tFL (19%) than in FL (5%, *P* = 0.05), and in non-GCB DLBCL (4%, *P* < 0.05) (Fig. 2 and Supplemental Tables S10-S11). Finally, the *IGHV3-30* gene, which was frequently used in tFL (13%), was completely absent from both types of DLBCL (*P* < 0.05). Similarly, the *IGHV4-34* gene was overrepresented in tFL relative to GCB DLBCL, in which it was never present (16 vs. 0%, *P* = 0.05) (Fig. 2 and Supplemental Tables S10–S11). Statistical significances were lost after Bonferroni correction due to the relative low number of cases in each of the groups.

### Effect of *IGHV* gene usage and mutational status on clinical outcomes

#### Clinical outcomes in whole FL series

In the whole series, after the median follow-up of 7.9 years (range: 0.6–21.4 years) for surviving patients, 48 patients had died. The 5- and 10-year OS were 82 and 70%, respectively, and the 5-year OS for low/intermediate-risk FLIPI was 92% (*P* < 0.01). Most of the treated patients, received R-ICT (60%) and the 5- and 10-year FFS were 51 and 29%, respectively. After induction therapy, 39 patients (40%) had achieved CR and 48 patients (54%) were in CR at 30 months (CR30).

We explored the use of *IGHV* subgroups and genes with clinical outcome. Two FL patients with *IGHV5* subgroup had poorer clinical course that the remaining patients and both died before the time of median follow-up. No correlations were observed between the *IGHV* gene usage and response or CR30, even taking into account only the 130 FL patients in whom the response was assessed by CT scan. No statistically significant differences were observed between the *IGHV* gene and FFS or OS.

There were four unmutated patients, all of whom had an intermediate/high-risk FLIPI status, although β2-microglobulin was always normal. Three of these patients received R-ICT but none was in CR at 30 months (data not shown). The three cases experienced an early treatment failure, leading to a shorter 5-year FFS than found in mutated FL cases (0% vs. 57%, *P* < 0.001). Two of these cases had died due to progression of their disease 29 and 40 months after initial diagnosis.

#### Risk of Histological Transformation

Considering only the 45 patients who developed clonally related HT, 18 patients had died after a median follow-up of 10.2 years for surviving patients (range: 1.2–21.4 years), giving a 5 and 10-year OS of 82 and 59%, respectively. The median TTT was 4.4 years after diagnosis (range: 0.1–19.6 years), with an estimated 84% of 10-year HT. Thirty-three (73%) patients received treatment as symptomatic FL. The other patients had never been treated until the date of HT into aggressive lymphoma. In this context, those who had never been treated (*n* = 12) had a higher 10-year HT (100 vs. 79%, *P* < 0.01) and shorter median TTT (2.9 vs. 6.6 years, *P* < 0.01) than those who received treatment as symptomatic FL (*n* = 33).

**Fig. 3:**
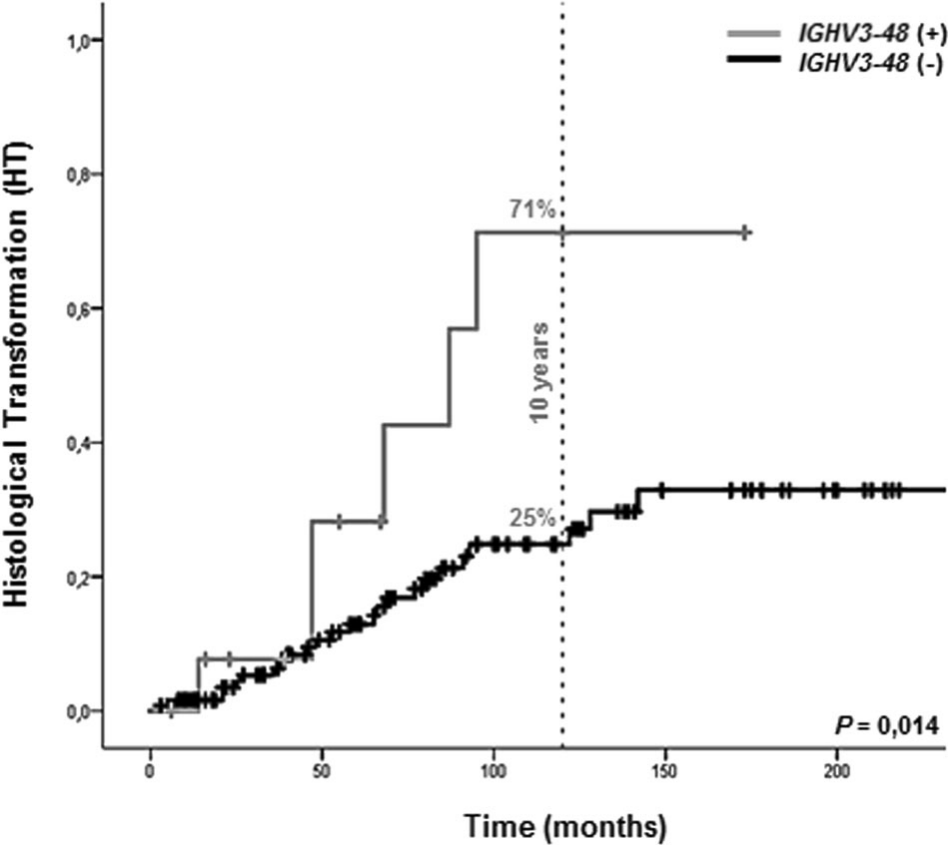
Kaplan–Meier analysis of 10-year HT by *IGHV3-48* gene usage. The absence and presence of *IGHV3-48* are depicted by black and grey lines, respectively. The vertical dashed line indicates 10-year follow-up

In the global series (*n* = 187), the 10-year HT was significantly higher for FL patients who never received treatment than for those who were treated (85 vs. 24%, *P* < 0.01). With respect to the *IGHV* gene, six out of 14 (43%) patients harboring the *IGHV3-48* gene at diagnosis experienced HT, resulting in a significantly higher 10-year HT than for those FL patients who had any other *IGHV* genes (71 vs. 25%, *P* < 0.05; Fig. 3). This result did not differ after excluding those cases with limited follow-up (*n* = 7 below 30 months, 5%; data not shown).

In the multivariate analysis, variables independently associated with longer 10-year HT were never treatment received (HR: 21.2, 95% CI: 6.3–70.7) and *IGHV3-48* gene usage (HR: 5.0, 95% CI: 1.5–17.2) (Table 3).

**Table 3:** Univariate and multivariate analysis of factors influencing cumulative incidence of histological transformation (HT)

Variable	HT (%) at 10 years	U (*p*-value)	M (*p*-value)	HR [95% CI]
FL grade				
I (reference)	26.8%	0.556	—	—
II	25.6%		0.976	1.0 [0.4–2.7]
IIIA	28.6%		0.126	3.7 [0.7–19.3]
FLIPI				
Low (reference)	27.2%	0.834	—	—
Intermediate	26.0%		0.393	0.6 [0.2–1.9]
High	20.1%		0.677	1.3 [0.4–4.1]
Never treated *(yes)*	85.3%	3.6 × 10^−7^	6.7 × 10^−7^	21.2 [6.3–70.7]
*IGHV3-48* gene *(yes)*	71.3%	0.014	0.010	5.0 [1.5–17.2]

*CI* confidence interval, *M* multivariate analysis, *FLIPI* follicular lymphoma international prognostic index, *HT* cumulative incidence of HT, *HR* hazard ratio, *U* univariate analysis

## Discussion

Although FL is the most frequent variant of indolent non-Hodgkin lymphoma, its ontogeny is still not well understood. Our study reports the largest FL series examined to date (*n* = 187) in which the use of *IGHV*, *IGHD* and *IGHJ* genes in the clonal BCR rearrangement has been extensively characterized. We also analyze of the potential association with clinical outcome and, for the first time, the risk of histological transformation (HT). We observed a biased use of certain *IGHV* genes, which provides new insight into the ontogeny of FL as part of B-cell differentiation. Thus, FL shows clear differences from other B-LPDs with regard to *IGHV* usage and SHM. Our findings suggest that certain antigens are involved in FL development through the stimulation of B-cells proliferation in cells expressing surface IG encoded by specific *IGHV* genes.

No differences were observed in the frequencies of *IGHV* subgroups between FL cells and CD5-/IgM+normal B-cells, although *IGHV1* frequency was slightly lower in the patient cohort^39^. These numbers mirrored those of other smaller series^14,25–27,45,46,47^. Conversely, significant differences were observed in the *IGHV* use compared with CD5-/IgM+normal B cells^39^. Thus, there was a strong bias towards higher gene usage of *IGHV4-34*, *IGHV3-23*, *IGHV3-48*, *IGHV3-30* and *IGHV3-21* in FL, these cases involving around half of the patient cohort. These results are similar to those previously reported, although *IGHV4-34* gene was more frequent in our series than in other FL series, probably due to differences in sample size or geographic distribution^48^. Compared with other B-LPDs, we also note a bias in *IGHV* gene usage in FL. The most interesting finding may be the high frequency of the *IGHV3-30* and *IGHV4-34* genes in tFL compared with their very low frequency or complete absence in GCB or non-GCB DLBCL^21,49,50^. The increased selection of *IGHV4-34* in tFL would favor a more immature cell of origin or with an extra-germinal center development. This information could be very helpful at diagnosis for distinguishing between DLBCL originating from histological transformation and true de novo DLBCL. Nevertheless, an analysis of a larger series is desirable in order to confirm this relationship. Taken together, these findings suggest that certain antigens participate in the lymphomagenesis of FL by stimulating the expression of surface IGs encoded by specific *IGHV* genes.

We analyzed the influence of the *IGHV* gene usage and SHM on clinical outcomes. None of the *IGHV* subgroups or genes appeared to be associated with clinical outcome, indicating that there is no relationship between *IGHV* usage and treatment requirement or response to therapy. The only relevant finding was that the two FL patients in the *IGHV5* subgroup had a short survival, which is consistent with a previous report^14^, although this is not sufficient to allow a definitive conclusion to be drawn. For the other *IGHV* genes, the low selection rate seen in FL precludes any reliable interpretation about their clinical significance. A larger series (probably of > 1000 cases) needs to be analyzed, as has recently been done with CLL and MM^23,51^. Something similar can be said in regard to *IGHV* mutational status, since only four of our cases had unmutated *IGHV*, consistent with the proportions in previous reports^46,52^. Nevertheless, all four unmutated cases had poor prognostic findings and short survival. A careful review of these four cases ruled out the presence of concomitant B-LPDs. The inclusion of several standard treatments with R-ICT has to be taken in consideration as a potential limitation of the present study.

In this report, we have analyzed for the first time the role of *IGHV* gene in histological transformation by analyzing 47 paired FL and tFL samples from the same patients. First, we confirmed the clonal relationship between each sample pair. Only two patients (4%) had a different clonal rearrangement between the two samples and were excluded from subsequent analyses. Consequently, 45 patients (24%) underwent clonally related HT into aggressive DLBCL. We observed that those FL patients in our cohort who were never treated had significantly higher 10y-HT than FL patients who received treatment, suggesting that early treatment is associated with a lower transformation risk, that is consistent with those of other studies^9,53^.

The *IGHV3-48* gene was associated with the risk of HT in the multivariate analysis, suggesting that it has a biological role in the transformation. Our FL series did not display a biased usage of *IGHV3-48* compared with other published FL series^14,27,46^, although *IGHV3-48* was significantly more frequent in HT than in non-transformed FL at 5 years (19 vs. 5%). Recently, the use of rituximab as first-line treatment in FL has been associated with a reduced risk of HT^53^. Since most *IGHV3-48* FL cases received R-ICT, the protective effect of rituximab on HT seems not to work so well for them. In addition, *IGHV3-48* FL cases were not associated to outcome probably due to some patients could be rescued by using rituximab-based therapy followed by auto-SCT to treat the transformation. Therefore, this finding implies that the *IGHV3-48* gene functions as a pathogenic factor involved in transformation, as seems to happen in CLL that bear *IGHV4-39* gene^54^.

In summary, in the largest FL series examined to date, a particular bias in *IGHV* gene selection towards the clonal BCR rearrangement was observed. The selection of the *IGHV3-48* gene seems to have a role in malignant transformation from FL to aggressive lymphoma. This argues in favor of the close monitoring of FL patients, examining *IGHV3-48* in anticipation of a potential transformation. We also found that *IGHV3-30* could contribute to the differential diagnostic process. Taken together, these results indicate a role for antigen selection in the development and transformation of FL, and suggest possibilities for future research into the biology of tFL.

## Supplementary information

Supplementary file
