# Supplementary material for: Immunoglobulin gene rearrangement IGHV3-48 is a predictive marker of histological transformation into aggressive lymphoma in follicular lymphomas
Source: Blood Cancer J. 2019 Jun 17;9(7):52. doi: 10.1038/s41408-019-0213-9 (PMC6579759; doi:10.1038/s41408-019-0213-9)
Supplement: Supplementary file 1 — Supplementary file [file 41408_2019_213_MOESM1_ESM.pdf]

Supplementary Figure 1. Flowchart of training cohort.

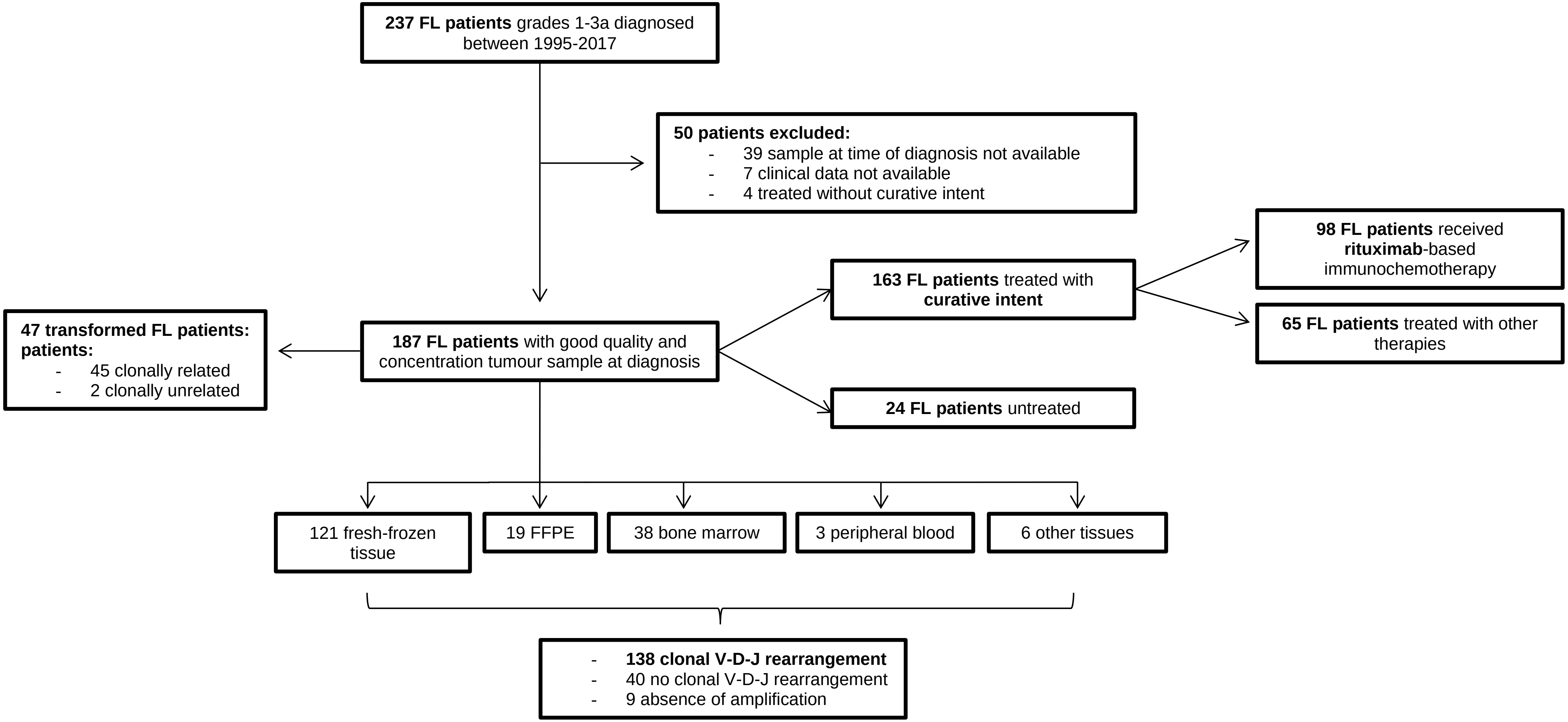

Supplementary Figure 2. (A) Homology of IGHV genes in training cohort. The boxplot shows percentage of homology according to IGHV subgroup in 130 sequences obtained from 138 patients studied. Horizontal lines represent median percentage of homology for the IGHV subgroup. (B) Unmutated (>98% homology) and mutated (<98% homology) frequency according to IGHV subgroup in 130 sequences studied.

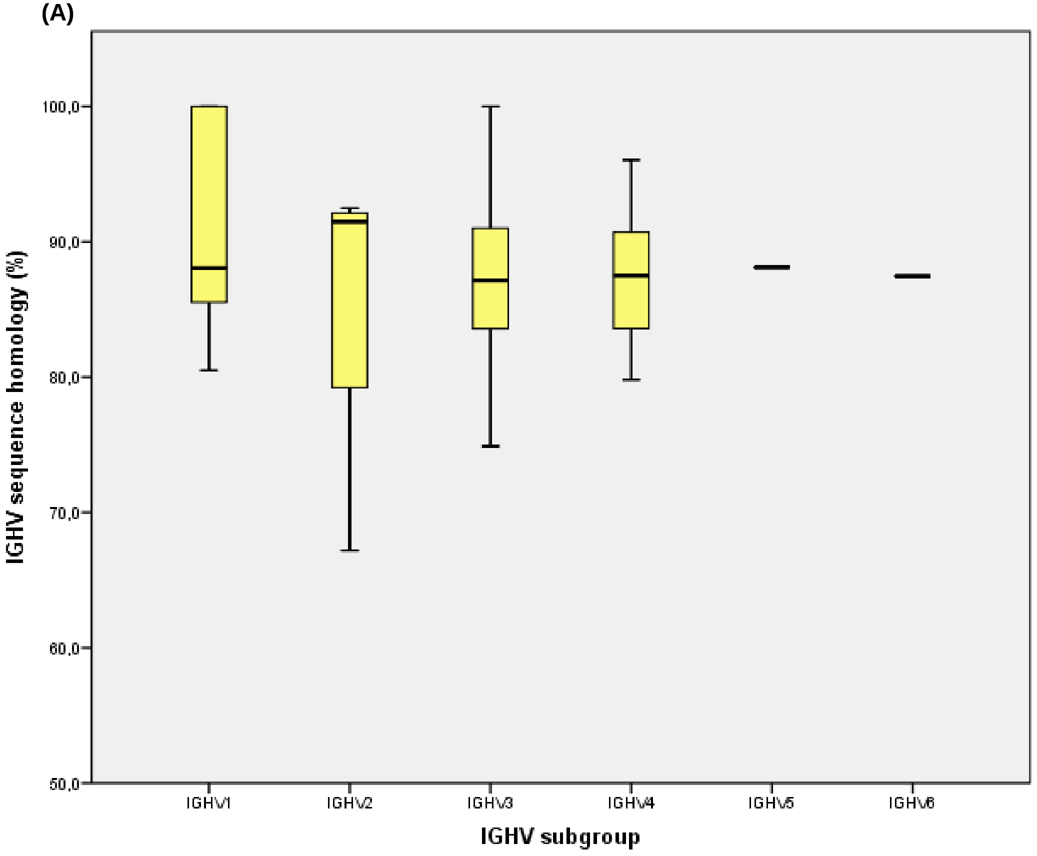

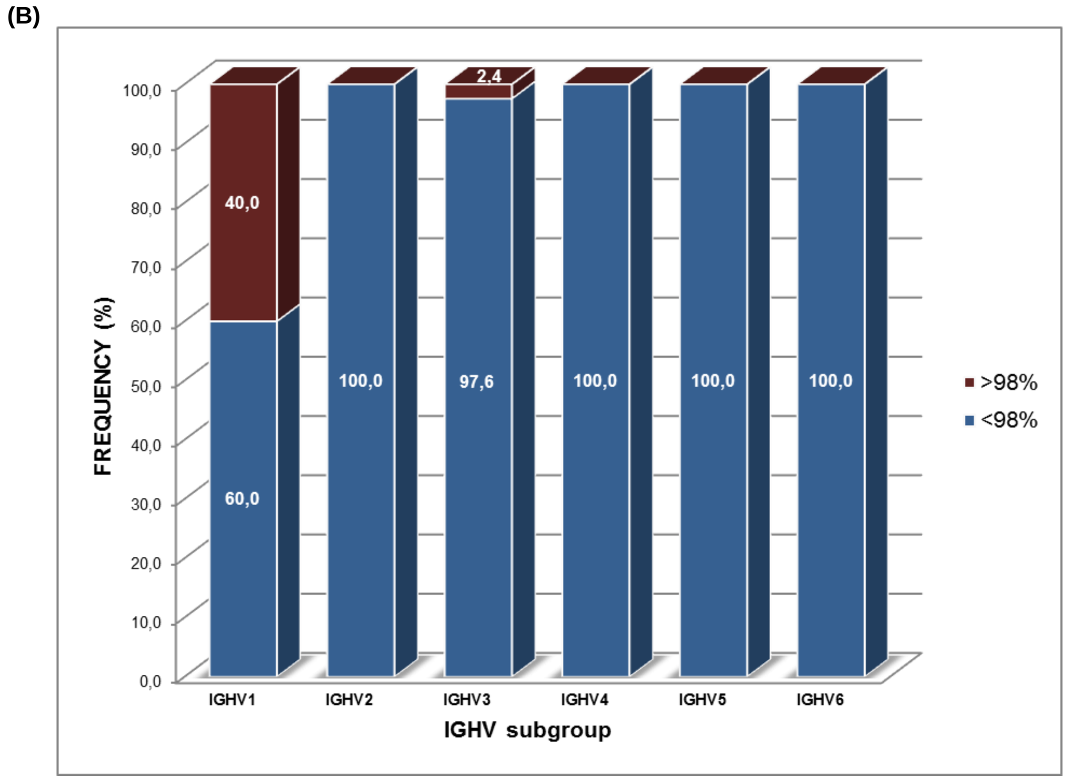

Table S1. Clinical characteristics of FL series included (larger cohort, n=187) vs. FL patients with clonal IGH rearrangements available (study cohort, n=138).

**Variable**	**Larger cohort n (%)**	**Study cohort n (%)**	***P***
**Age, years (median, range)**	58 (19-87)	59 (19-87)	ns
**Sex F/M**	99 (53)/88 (47)	79 (57)/59 (43)	ns
**Histological grade***			
I	84 (48.8)	60 (44.8)	ns
II	67 (39)	58 (43.3)	ns
IIIA	21 (12.2)	12 (9.0)	ns
**FLIPI***			
0-1 (Low risk)	49 (32.9)	32 (27.4)	ns
2 (Intermediate risk)	46 (30.9)	39 (33.3)	ns
3-5 (High risk)	54 (36.2)	46 (39.3)	ns
**Ann Arbor***			
I	24 (14.2)	12 (9.2)	ns
II	18 (10.7)	13 (10.0)	ns
III	18 (10.7)	14 (10.8)	ns
IV	109 (64.5)	91 (70.0)	ns
**First-line therapy**			
Never treated	24 (12.8)	16 (11.6)	ns
Rituximab-based ICT	98 (52.4)	80 (58.0)	ns
R-CHOP	68 (69.4)	53 (66.3)	ns
R-Bendamustine	11 (11.2)	8 (10.0)	ns
R-CVP	3 (3.1)	3 (3.7)	ns
Others	16 (16.3)	16 (20.0)	ns
CT without rituximab	48 (25.7)	34 (24.6)	ns
CHOP	35 (72.9)	25 (73.5)	ns
Fludarabine-based	3 (6.3)	2 (5.9)	ns
Others	10 (20.8)	7 (20.6)	ns
Radiotherapy alone or with rituximab	13 (7)	6 (4.3)	ns
Rituximab alone	4 (2.1)	2 (1.4)	ns
**Maintenance with rituximab***	73 (39)	60 (54.1)	ns
**Response after induction therapy**			
CR	87 (53.7)	61 (50.4)	ns
PR	63 (38.9)	51 (42.1)	ns
NR/Failure	12 (7.4)	9 (7.4)	ns

***Larger cohort:** Histological grade was available for 172 (92%) patients; FLIPI was available for 149 (80%) patients; Ann Arbor was available for 169 (90%) patients; Maintenance was calculated for 147 (79%) patients.

Study cohort: Histological grade was available for 130 (94%) patients; FLIPI was available for 117 (85%) patients; Ann Arbor was available for 130 (94%) patients; Maintenance was calculated for 111 (80%) patients.

ICT: Immunochemotherapy; R, rituximab; CHOP, cyclophosphamide/doxorubicin/vincristine/prednisone; CVP, cyclophosphamide/vincristine/prednisone; QT: Chemotherapy; CR: Complete response; PR: Partial response; NR: No response; FLIPI: FL International Prognosis Index.

Table S2. Frequencies of IGHV, IGHD and IGHJ subgroups in the clonal V-D-J rearrangements of follicular lymphoma.

IGH subgroup	Frequency, n	Frequency, %
**IGHV1**	7	5,1
**IGHV2**	4	2,9
**IGHV3**	87	63,0
**IGHV4**	37	26,8
**IGHV5**	2	1,4
**IGHV6**	1	0,7
**IGHV7**	0	0,0
**Totals**	**138**	**100,0**

IGH subgroup	Frequency, n	Frequency, %
**IGHD1**	12	9,6
**IGHD2**	31	24,8
**IGHD3**	46	36,8
**IGHD4**	15	12,0
**IGHD5**	5	4,0
**IGHD6**	16	12,8
**IGHD7**	0	0,0
**Totals**	**125**	**100,0**

IGH subgroup	Frequency, n	Frequency, %
**IGHJ1**	1	0,8
**IGHJ2**	3	2,4
**IGHJ3**	15	11,9
**IGHJ4**	60	47,6
**IGHJ5**	22	17,5
**IGHJ6**	25	19,8
**Totals**	**126**	**100,0**

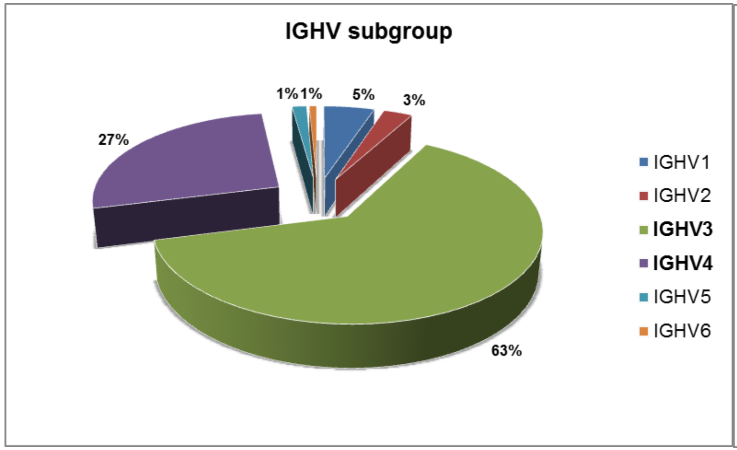

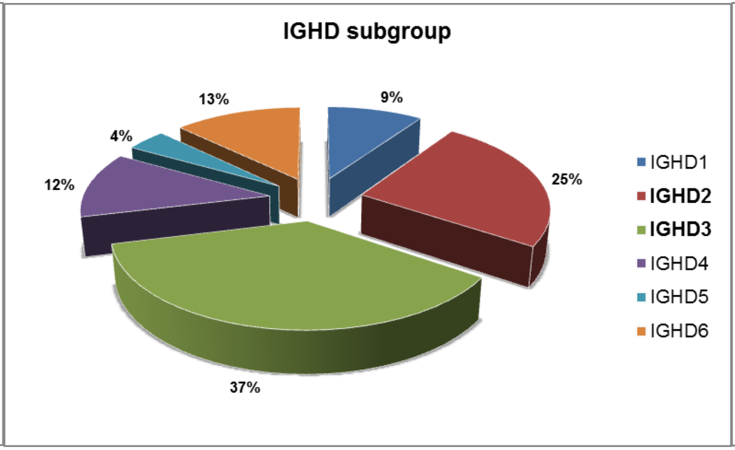

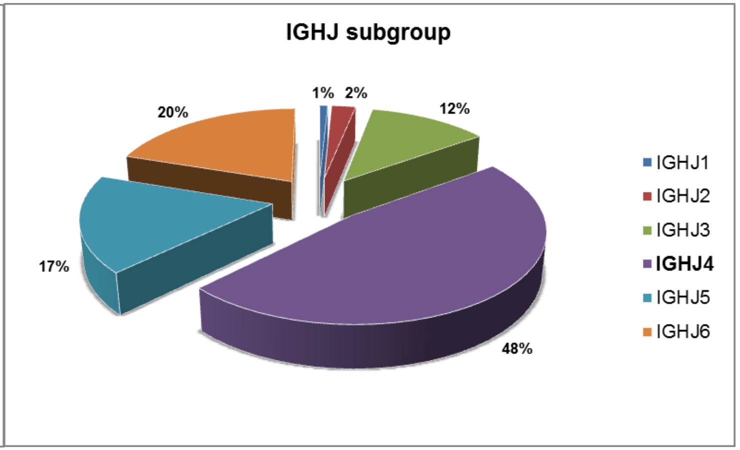

Table S3. IGHV gene repertoire of the present series.

IGHV gene	Frequency, n	Frequency, %	IGHV gene	Frequency, n	Frequency, %	IGHV gene	Frequency, n	Frequency, %
**IGHV1-2**	0	0,0	**IGHV3-30**	13	9,4	**IGHV4-38-2**	0	0,0
**IGHV1-3**	0	0,0	**IGHV3-30-3**	1	0,7	**IGHV4-39**	6	4,3
**IGHV1-8**	1	0,7	**IGHV3-30-5**	0	0,0	**IGHV4-55**	1	0,7
**IGHV1-18**	2	1,4	**IGHV3-33**	5	3,6	**IGHV4-59**	1	0,7
**IGHV1-24**	0	0,0	**IGHV3-43**	0	0,0	**IGHV4-61**	5	3,6
**IGHV1-45**	0	0,0	**IGHV3-43D**	0	0,0	**IGHV5-10-1**	1	0,7
**IGHV1-46**	1	0,7	**IGHV3-48**	14	10,1	**IGHV5-51**	1	0,7
**IGHV1-58**	0	0,0	**IGHV3-49**	0	0,0	**IGHV6-1**	1	0,7
**IGHV1-69**	3	2,2	**IGHV3-53**	2	1,4	**IGHV7-4-1**	0	0,0
**IGHV1-69-2**	0	0,0	**IGHV3-64**	2	1,4	**Totals**	**138**	**100,0**
**IGHV1-69D**	0	0,0	**IGHV3-64D**	0	0,0			
**IGHV2-5**	2	1,4	**IGHV3-66**	1	0,7			
**IGHV2-26**	1	0,7	**IGHV3-72**	1	0,7			
**IGHV2-70**	1	0,7	**IGHV3-73**	2	1,4			
**IGHV2-70D**	0	0,0	**IGHV3-74**	2	1,4			
**IGHV3-7**	6	4,3	**IGHV3-NL1**	0	0,0			
**IGHV3-9**	0	0,0	**IGHV4-4**	1	0,7			
**IGHV3-11**	3	2,2	**IGHV4-28**	0	0,0			
**IGHV3-13**	1	0,7	**IGHV4-30**	2	1,4			
**IGHV3-15**	4	2,9	**IGHV4-30-1**	0	0,0			
**IGHV3-20**	1	0,7	**IGHV4-30-2**	1	0,7			
**IGHV3-21**	10	7,2	**IGHV4-30-4**	0	0,0			
**IGHV3-23**	19	13,8	**IGHV4-31**	0	0,0			
**IGHV3-23D**	0	0,0	**IGHV4-34**	20	14,5			

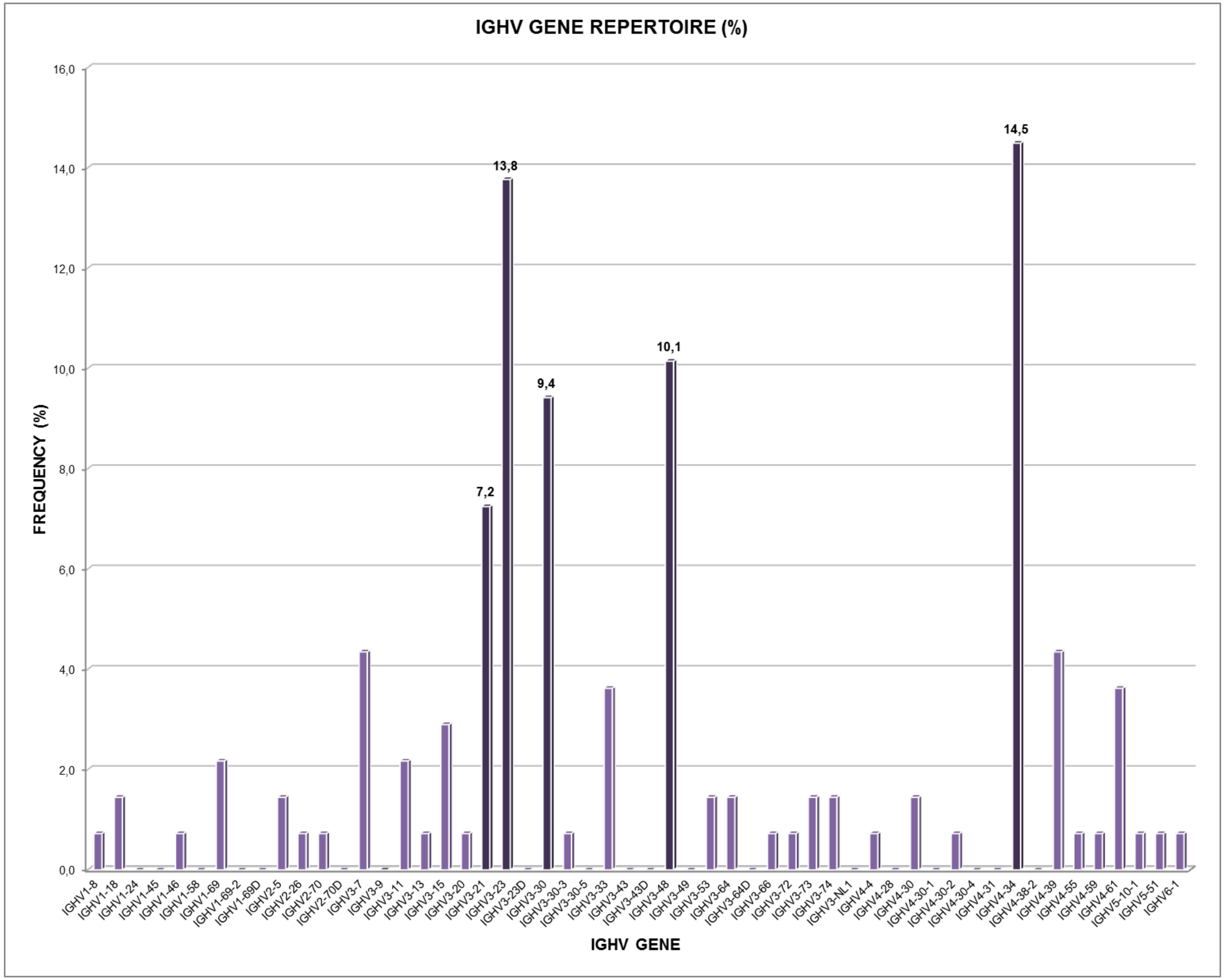
IGHV GENE REPERTOIRE (%)
FREQUENCY (%)
16,0
14,0
12,0
10,0
8,0
6,0
4,0
2,0
0,0
14,5
13,8
10,1
9,4
7,2
IGHV1-8
IGHV1-18
IGHV1-24
IGHV1-45
IGHV1-46
IGHV1-58
IGHV1-69
IGHV1-69-2
IGHV1-69D
IGHV2-5
IGHV2-26
IGHV2-70
IGHV2-70D
IGHV3-7
IGHV3-9
IGHV3-11
IGHV3-13
IGHV3-15
IGHV3-20
IGHV3-21
IGHV3-23
IGHV3-23D
IGHV3-30
IGHV3-30-3
IGHV3-30-5
IGHV3-33
IGHV3-43
IGHV3-43D
IGHV3-48
IGHV3-49
IGHV3-53
IGHV3-64
IGHV3-64D
IGHV3-66
IGHV3-72
IGHV3-73
IGHV3-74
IGHV3-NL1
IGHV4-4
IGHV4-28
IGHV4-30
IGHV4-30-1
IGHV4-30-2
IGHV4-30-4
IGHV4-31
IGHV4-34
IGHV4-38-2
IGHV4-39
IGHV4-55
IGHV4-59
IGHV4-61
IGHV5-10-1
IGHV5-51
IGHV6-1
IGHV GENE

Table S4. IGHD gene repertoire of the present series.

IGHD gene	Frequency, n	Frequency, %
**IGHD1-1**	4	3,2
**IGHD1-7**	1	0,8
**IGHD1-14**	0	0,0
**IGHD1-20**	3	2,4
**IGHD1-26**	4	3,2
**IGHD2-2**	9	7,2
**IGHD2-8**	4	3,2
**IGHD2-15**	8	6,4
**IGHD2-21**	10	8,0
**IGHD3-3**	6	4,8
**IGHD3-9**	3	2,4
**IGHD3-10**	15	12,0
**IGHD3-16**	10	8,0
**IGHD3-22**	12	9,6
**IGHD4-4**	0	0,0
**IGHD4-11**	5	4,0
**IGHD4-17**	4	3,2
**IGHD4-23**	6	4,8
**IGHD5-5**	0	0,0
**IGHD5-12**	3	2,4
**IGHD5-18**	1	0,8
**IGHD5-24**	1	0,8
**IGHD6-6**	5	4,0
**IGHD6-13**	3	2,4
**IGHD6-19**	6	4,8
**IGHD6-25**	2	1,6
**IGHD7-27**	0	0,0
**Totals**	**125**	**100,0**

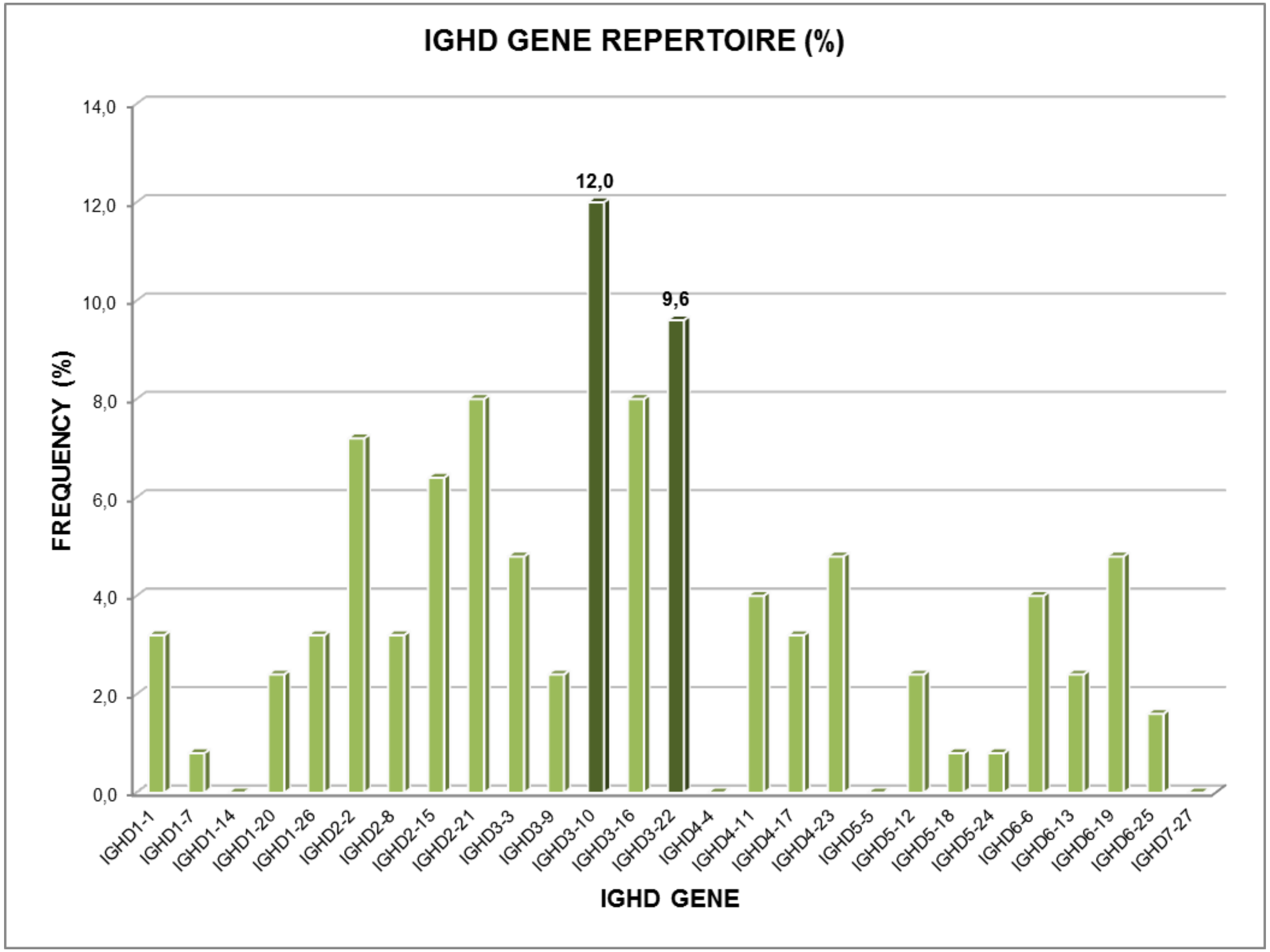

IGHD GENE REPERTOIRE (%)
FREQUENCY (%)
14,0
12,0
10,0
8,0
6,0
4,0
2,0
0,0
12,0
9,6
IGHD1-1
IGHD1-7
IGHD1-14
IGHD1-20
IGHD1-26
IGHD2-2
IGHD2-8
IGHD2-15
IGHD2-21
IGHD3-3
IGHD3-9
IGHD3-10
IGHD3-16
IGHD3-22
IGHD4-4
IGHD4-11
IGHD4-17
IGHD4-23
IGHD5-5
IGHD5-12
IGHD5-18
IGHD5-24
IGHD6-6
IGHD6-13
IGHD6-19
IGHD6-25
IGHD7-27
IGHD GENE

Table S5. VH CDR3 length distribution for the IGHV subgroup and most frequent IGHV gene in FL (n=113).

	IGHV1		IGHV2		IGHV3		IGHV4		IGHV5		IGHV6	
**VH CDR3 length**	**No**	**%**	**No**	**%**	**No**	**%**	**No**	**%**	**No**	**%**	**No**	**%**
**8**	0	0,0	0	0,0	1	1,4	0	0,0	0	0,0	0	0,0
**9**	0	0,0	0	0,0	1	1,4	0	0,0	0	0,0	0	0,0
**10**	0	0,0	0	0,0	4	5,6	1	3,1	0	0,0	0	0,0
**11**	2	50,0	0	0,0	5	7,0	0	0,0	0	0,0	0	0,0
**12**	0	0,0	0	0,0	5	7,0	0	0,0	0	0,0	0	0,0
**13**	0	0,0	0	0,0	5	7,0	3	9,4	1	100,0	0	0,0
**14**	0	0,0	1	25,0	4	5,6	4	12,5	0	0,0	0	0,0
**15**	0	0,0	0	0,0	7	9,9	3	9,4	0	0,0	0	0,0
**16**	0	0,0	0	0,0	11	15,5	11	34,4	0	0,0	0	0,0
**17**	0	0,0	1	25,0	10	14,1	3	9,4	0	0,0	0	0,0
**18**	1	25,0	0	0,0	3	4,2	1	3,1	0	0,0	1	100,0
**19**	0	0,0	0	0,0	3	4,2	2	6,3	0	0,0	0	0,0
**20**	0	0,0	0	0,0	4	5,6	1	3,1	0	0,0	0	0,0
**21**	0	0,0	0	0,0	1	1,4	1	3,1	0	0,0	0	0,0
**22**	0	0,0	0	0,0	1	1,4	0	0,0	0	0,0	0	0,0
**23**	0	0,0	2	50,0	0	0,0	0	0,0	0	0,0	0	0,0
**25**	0	0,0	0	0,0	2	2,8	0	0,0	0	0,0	0	0,0
**26**	1	25,0	0	0,0	1	1,4	1	3,1	0	0,0	0	0,0
**27**	0	0,0	0	0,0	1	1,4	1	3,1	0	0,0	0	0,0
**28**	0	0,0	0	0,0	2	2,8	0	0,0	0	0,0	0	0,0
**Totals**	**4**	100,0	**4**	100,0	**71**	100,0	**32**	100,0	**1**	100,0	**1**	100,0
**Mean**	**16,5**		**19,3**		**16,0**		**16,5**		**13,0**		**18,0**	
**Median**	**14,5**		**20,0**		**16,0**		**16,0**		**13,0**		**18,0**	

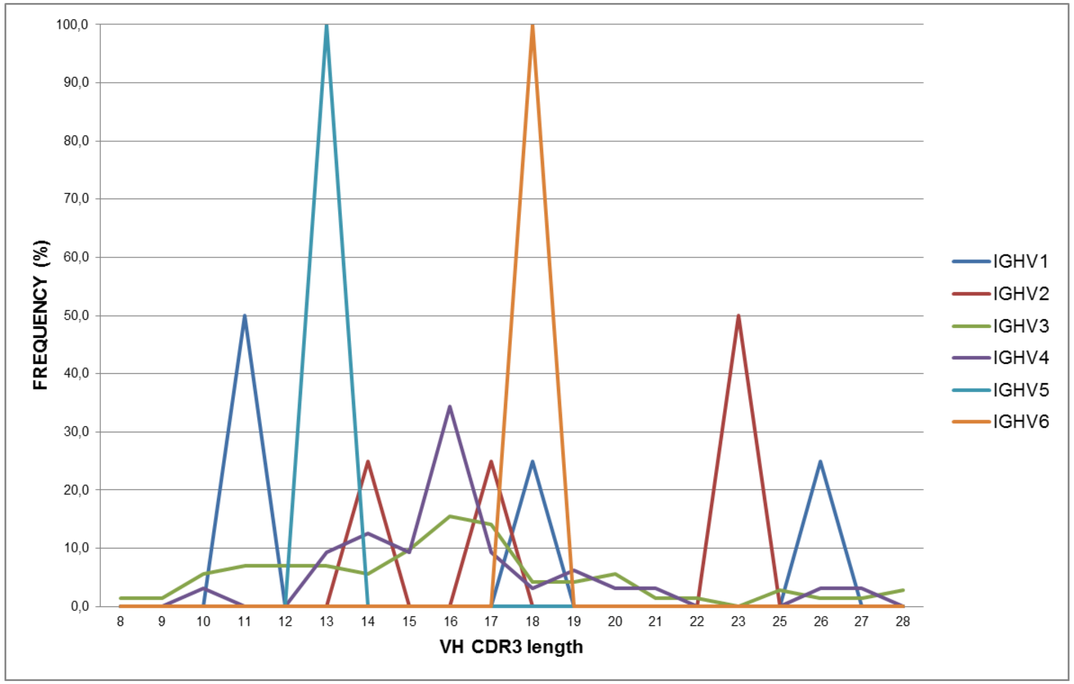

FREQUENCY (%)
100,0
90,0
80,0
70,0
60,0
50,0
40,0
30,0
20,0
10,0
0,0
8 9 10 11 12 13 14 15 16 17 18 19 20 21 22 23 25 26 27 28
VH CDR3 length
IGHV1
IGHV2
IGHV3
IGHV4
IGHV5
IGHV6

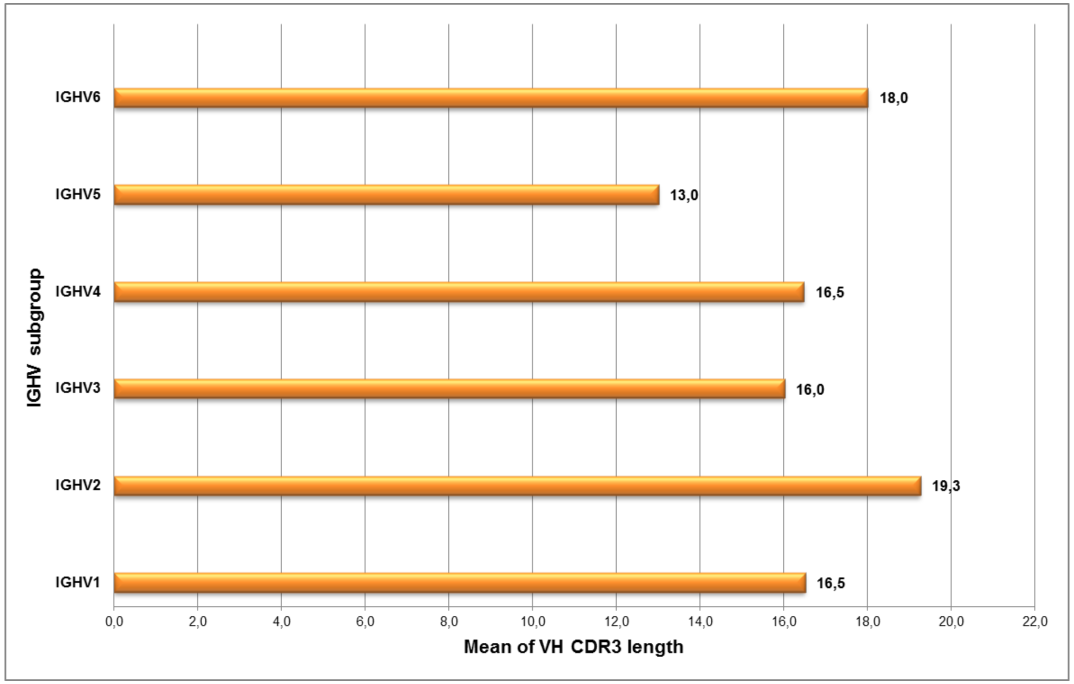

IGHV subgroup
IGHV6
18,0
IGHV5
13,0
IGHV4
16,5
IGHV3
16,0
IGHV2
19,3
IGHV1
16,5
0,0 2,0 4,0 6,0 8,0 10,0 12,0 14,0 16,0 18,0 20,0 22,0
Mean of VH CDR3 length

	IGHV3-21		IGHV3-23		IGHV3-30		IGHV3-48		IGHV4-34	
**VH CDR3 length**	**No**	**%**	**No**	**%**	**No**	**%**	**No**	**%**	**No**	**%**
**8**	0	0,0	0	0,0	0	0,0	0	0,0	0	0,0
**9**	0	0,0	1	6,7	0	0,0	0	0,0	0	0,0
**10**	1	16,7	0	0,0	0	0,0	1	8,3	1	5,3
**11**	2	33,3	0	0,0	0	0,0	0	0,0	0	0,0
**12**	0	0,0	1	6,7	1	10,0	0	0,0	0	0,0
**13**	0	0,0	1	6,7	0	0,0	2	16,7	1	5,3
**14**	1	16,7	1	6,7	1	10,0	1	8,3	3	15,8
**15**	0	0,0	4	26,7	1	10,0	0	0,0	1	5,3
**16**	1	16,7	2	13,3	1	10,0	2	16,7	7	36,8
**17**	0	0,0	2	13,3	2	20,0	2	16,7	2	10,5
**18**	0	0,0	1	6,7	0	0,0	0	0,0	1	5,3
**19**	0	0,0	1	6,7	0	0,0	2	16,7	1	5,3
**20**	1	16,7	0	0,0	1	10,0	2	16,7	0	0,0
**21**	0	0,0	0	0,0	0	0,0	0	0,0	1	5,3
**22**	0	0,0	0	0,0	0	0,0	0	0,0	0	0,0
**23**	0	0,0	0	0,0	0	0,0	0	0,0	0	0,0
**25**	0	0,0	0	0,0	2	20,0	0	0,0	0	0,0
**26**	0	0,0	1	6,7	0	0,0	0	0,0	0	0,0
**27**	0	0,0	0	0,0	0	0,0	0	0,0	1	5,3
**28**	0	0,0	0	0,0	1	10,0	0	0,0	0	0,0
**Totals**	**6**	100,0	**15**	100,0	**10**	100,0	**12**	100,0	**19**	100,0
**Mean**	**13,7**		**15,8**		**18,9**		**16,2**		**16,4**	
**Median**	**12,5**		**15,0**		**17,0**		**15,5**		**16,0**	

	All minus IGHV3-21		All minus IGHV3-23		All minus IGHV3-30		All minus IGHV3-48		All minus IGHV4-34		All minus more frequent IGHV gene	
**VH CDR3 length**	**No**	**%**	**No**	**%**	**No**	**%**	**No**	**%**	**No**	**%**	**No**	**%**
**8**	1	0,9	1	1,0	1	1,0	1	1,0	1	1,1	1	2,0
**9**	1	0,9	0	0,0	1	1,0	1	1,0	1	1,1	0	0,0
**10**	4	3,7	5	5,1	5	4,9	4	4,0	4	4,3	2	3,9
**11**	5	4,7	7	7,1	7	6,8	7	6,9	7	7,4	5	9,8
**12**	5	4,7	4	4,1	4	3,9	5	5,0	5	5,3	3	5,9
**13**	9	8,4	8	8,2	9	8,7	7	6,9	8	8,5	5	9,8
**14**	8	7,5	8	8,2	8	7,8	8	7,9	6	6,4	2	3,9
**15**	10	9,3	6	6,1	9	8,7	10	9,9	9	9,6	4	7,8
**16**	21	19,6	20	20,4	21	20,4	20	19,8	15	16,0	9	17,6
**17**	14	13,1	12	12,2	12	11,7	12	11,9	12	12,8	6	11,8
**18**	6	5,6	5	5,1	6	5,8	6	5,9	5	5,3	4	7,8
**19**	5	4,7	4	4,1	5	4,9	3	3,0	4	4,3	1	2,0
**20**	4	3,7	5	5,1	4	3,9	3	3,0	5	5,3	1	2,0
**21**	2	1,9	2	2,0	2	1,9	2	2,0	1	1,1	1	2,0
**22**	1	0,9	1	1,0	1	1,0	1	1,0	1	1,1	1	2,0
**23**	2	1,9	2	2,0	2	1,9	2	2,0	2	2,1	2	3,9
**25**	2	1,9	2	2,0	0	0,0	2	2,0	2	2,1	0	0,0
**26**	3	2,8	2	2,0	3	2,9	3	3,0	3	3,2	2	3,9
**27**	2	1,9	2	2,0	2	1,9	2	2,0	1	1,1	1	2,0
**28**	2	1,9	2	2,0	1	1,0	2	2,0	2	2,1	1	2,0
**Totals**	**107**	100,0	**98**	100,0	**103**	100,0	**101**	100,0	**94**	100,0	**51**	100,0
**Mean**	**16,4**		**16,3**		**16,0**		**16,3**		**16,2**		**16,2**	
**Median**	**16,0**		**16,0**		**16,0**		**16,0**		**16,0**		**16,0**	

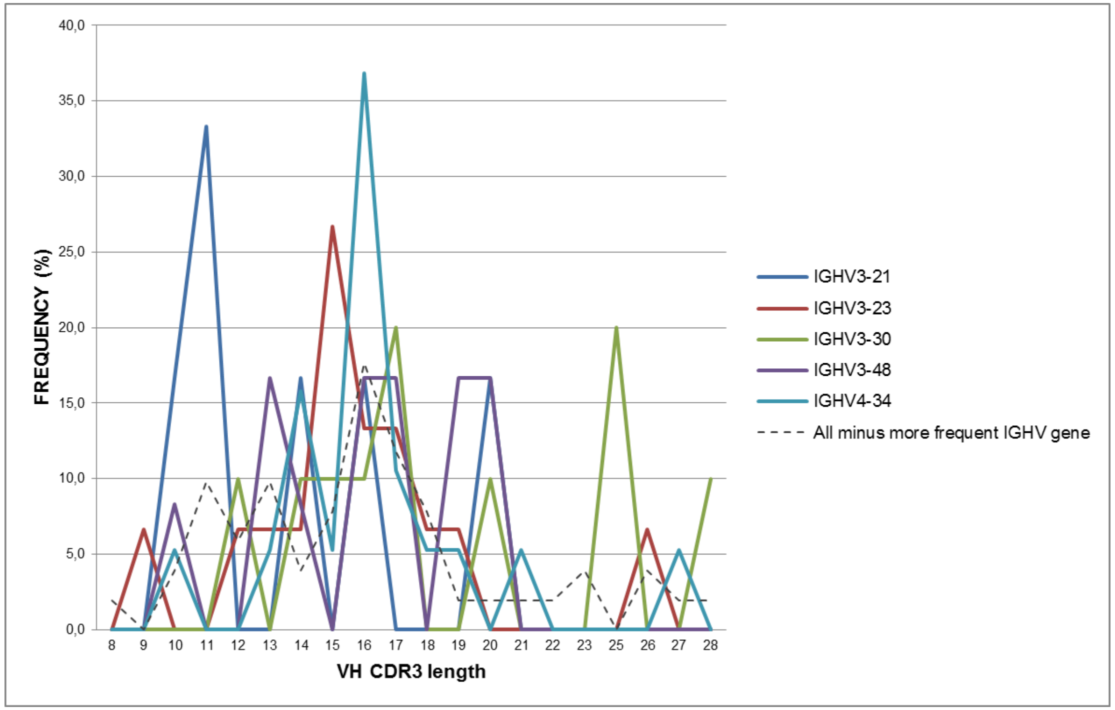
40,0
35,0
30,0
25,0
20,0
15,0
10,0
5,0
0,0
FREQUENCY (%)
8 9 10 11 12 13 14 15 16 17 18 19 20 21 22 23 25 26 27 28
VH CDR3 length
IGHV3-21
IGHV3-23
IGHV3-30
IGHV3-48
IGHV4-34
All minus more frequent IGHV gene

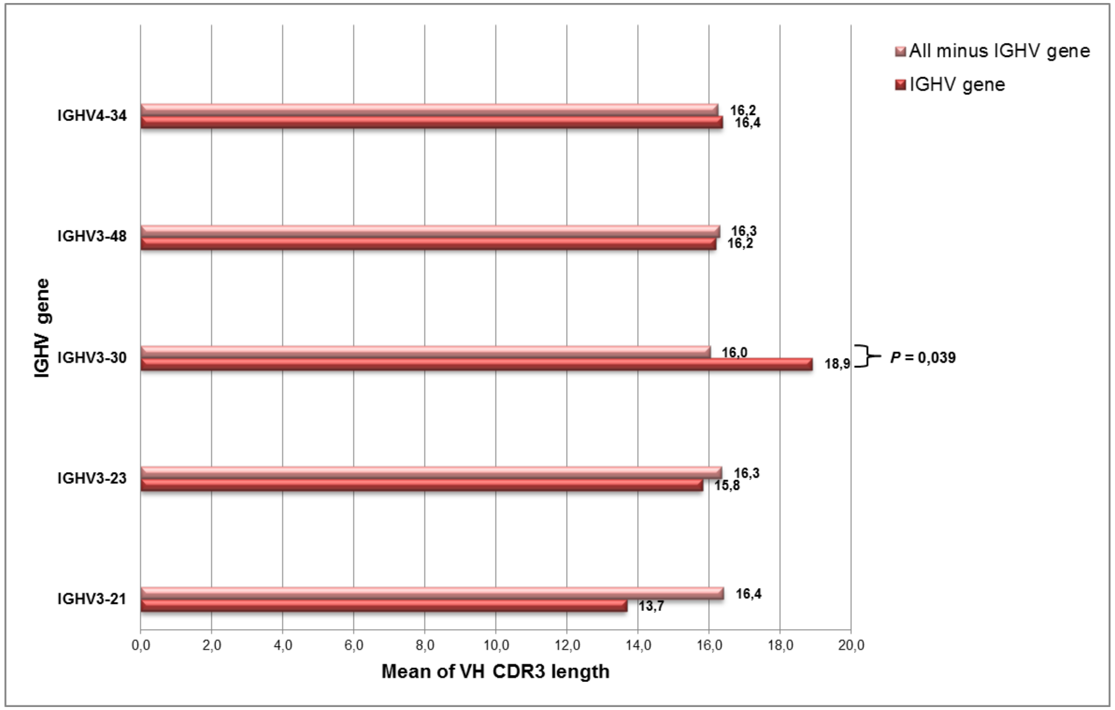
All minus IGHV gene
IGHV gene
IGHV4-34
16,2
16,4
IGHV3-48
16,3
16,2
IGHV3-30
16,0
18,9
P = 0,039
IGHV3-23
16,3
15,8
IGHV3-21
16,4
13,7
IGHV gene
0,0 2,0 4,0 6,0 8,0 10,0 12,0 14,0 16,0 18,0 20,0
Mean of VH CDR3 length

Table S6. Comparison of the IGHV, IGHD, and IGHJ subgroups and significantly different IGHV in our series of FL with those reported for B-LPDs and normal B cells.

IGH	FL (n=138)		DLBCL (n=103)[1]		*P*	MCL (n=807)[2]		*P*	B-CLL (n=7596)[3]		*P*	SMZL (n=133)[4]		*P*	HCL (n=102)[5]		*P*	MM (n=270)[6]		*P*	WM (n=58)[7]		*P*	CD5-/IgM+ (n=206)[8]		*P*
	n	%	n	%		n	%		n	%		n	%		n	%		n	%		n	%		n	%	
**IGHV**																										
1	7	5,1	17	16,5	0,0043*	125	15,5	0,0005*	1807	23,8	0,0001*	40	30,1	0,0001*	15	14,7	0,0129	42	15,6	0,0019*	4	6,9	0,7348	27	13,1	0,0161
2	4	2,9	2	1,9	1,0000	12	1,5	0,2732	255	3,4	1,0000	1	0,8	0,3708	3	2,9	1,0000	17	6,3	0,1625	0	0	0,3211	4	1,9	0,7184
3	87	63	45	43,7	0,0039*	416	51,6	0,0128	3658	48,2	0,0006*	66	49,6	0,0280	55	53,9	0,1843	132	48,9	0,0086*	44	75,9	0,0972	111	53,9	0,0964
4	37	26,8	34	33	0,3195	208	25,8	0,8336	1567	20,6	0,0892	23	17,3	0,0786	24	23,5	0,6532	55	20,4	0,1682	8	13,8	0,0621	51	24,8	0,7060
5	2	1,4	4	3,9	0,4062	41	5,1	0,0737	192	2,5	0,5871	3	2,3	0,6795	1	1	1,0000	21	7,8	0,0065*	1	1,7	1,0000	6	2,9	0,4832
6	1	0	1	1	1,0000	5	0,6	1,0000	88	1,2	1,0000	0	0	1,0000	3	2,9	0,3146	3	1,1	1,0000	1	1,7	0,5053	5	2,4	0,4080
7	0	0	0	0	1,0000	0	0	1,0000	29	0,4	1,0000	0	0	1,0000	1	1	0,4250	0	0	1,0000	0	0	1,0000	2	1	0,5181
3-21	10	7,2	5	4,8	0,5924	133	16,5	0,0044	356	4,7	0,1556	4	3	0,1692	3	3,1	0,1636	4	1,5	0,0067	3	5,2	0,7586	0	0	0,0001*
3-23	19	13,8	10	9,7	0,4246	60	7,4	0,0189	647	8,5	0,0442	24	18	0,4062	17	18,1	0,5853	13	5	0,0029	17	29,3	0,0148	25	12,1	0,7423
3-30	13	9,4	0	0	0,0008*	28	3,5	0,0048	416	5,5	0,0574	8	6	0,3657	8	8,5	0,8182	27	10	1,0000	5	8,6	1,0000	12	5,8	0,2123
3-48	14	10,1	6	5,8	0,2496	29	3,6	0,0028	295	3,9	0,0012	5	3,8	0,0553	5	5,3	0,1545	5	1,8	0,0003*	0	0	0,0116	9	4,4	0,0462
4-34	20	14,5	16	15,5	0,8564	118	14,6	1,0000	673	8,9	0,0333	10	7,5	0,0818	7	7,4	0,0968	2	0,9	0,0001*	0	0	0,0012	8	3,9	0,0005*
**IGHD**																										
1	12	9,6	11	10,7		86	10,6		618	8,2		6	5,2		13	12,7		20	7,6		16	27,3		13	6,4	
2	31	24,8	23	21,4		140	17,4		1471	19,6		18	15,5		13	12,7		65	24,2		3	4,5		44	21,2	
3	46	36,8	37	35,9		276	34,2		3022	40,3		54	46,6		42	41,2		70	25,8		10	18,2		77	37,2	
4	15	12	6	5,8		69	8,5		476	6,3		9	7,8		7	6,9		29	10,6		8	13,6		22	10,9	
5	5	4	9	9,7		67	8,3		662	8,8		11	9,5		7	6,9		37	13,6		5	9,1		21	10,3	
6	16	12,8	15	14,6		164	20,3		1195	15,9		18	15,5		19	18,6		33	12,1		16	27,3		21	10,3	
7	0	0	2	1,9		5	0,6		54	0,7		0	0		1	1		16	6,1		0	0		8	3,8	
**IGHJ**																										
1	1	0,8	4	3,9		5	0,6		137	1,8		2	1,5		3	2,9		4	1,4		2	4,4		2	1	
2	3	2,4	5	4,8		28	3,5		171	2,3		0	0		4	3,9		4	1,4		5	8,9		4	2	
3	15	11,9	13	12,6		61	7,6		753	9,9		19	14,3		10	9,8		48	17,8		8	13,3		17	8	
4	60	47,6	46	44,7		353	43,7		3289	13,3		50	37,6		47	46,1		144	53,4		21	35,6		113	55	
5	22	17,5	13	12,6		121	15		796	10,5		26	19,5		13	12,7		22	8,2		8	13,3		21	10	
6	25	19,8	22	21,4		239	29,6		2450	32,3		36	27,1		25	24,5		48	17,8		14	24,4		49	24	

References: **1**-Diffuse Large B-Cell Lymphoma, Sebastian et al, American Journal of Pathology 2012; **2**-Mantle cell lymphoma, Hadzidimitriou et al, Blood 2011; **3**-B-cell Chronic lymphocytic leukemia, Agathangelidis et al, Blood 2012; **4**-Splenic marginal zone lymphoma, Zibellini et al, Haematologica 2010; **5**-Hairy cell leukemia, Arons et al, Blood 2011; **6**-Multiple myeloma, González et al, Blood 2007; **7**-Waldenström macroglobulinemia, Martín-Jiménez et al, Haematologica 2007; **8**-CD5-/IgM+ B-cells, Brezinschek et al, Journal of Clinical Investigation 1997. *****Statistically significant after Bonferroni correction ($Pc < 0.05$).

Table S7. Frequencies of IGHV, IGHD and IGHJ subgroups in the clonal V-D-J rearrangements of transformed follicular lymphoma (tFL).

IGH subgroup	Frequency, n	Frequency, %
IGHV1	1	3,2
IGHV2	0	0,0
IGHV3	23	74,2
IGHV4	6	19,4
IGHV5	1	3,2
IGHV6	0	0,0
IGHV7	0	0,0
**Totals**	**31**	**100,0**

IGH subgroup	Frequency, n	Frequency, %
IGHD1	3	12,0
IGHD2	8	32,0
IGHD3	8	32,0
IGHD4	2	8,0
IGHD5	0	0,0
IGHD6	4	16,0
IGHD7	0	0,0
**Totals**	**25**	**100,0**

IGH subgroup	Frequency, n	Frequency, %
IGHJ1	0	0,0
IGHJ2	1	3,8
IGHJ3	3	11,5
IGHJ4	12	46,2
IGHJ5	4	15,4
IGHJ6	6	23,1
**Totals**	**26**	**100,0**

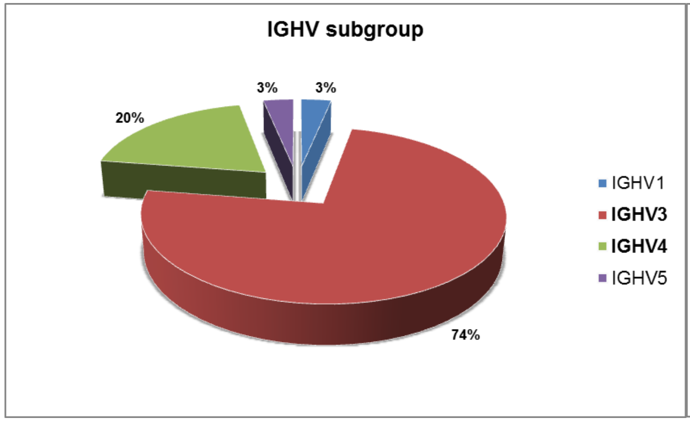

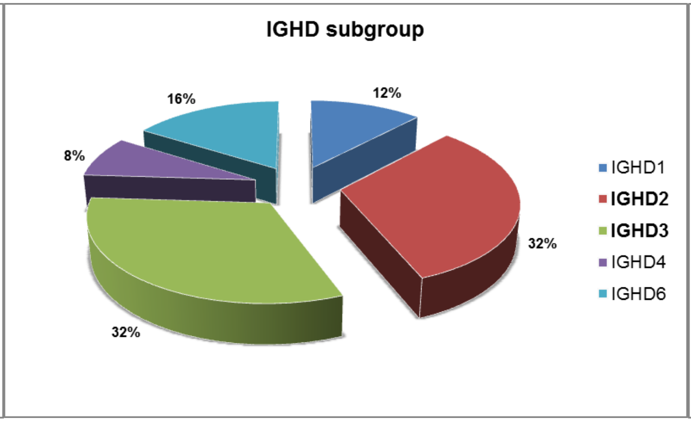

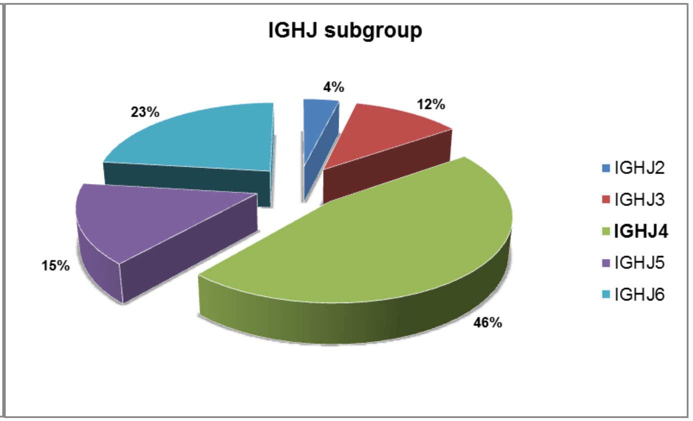

Table S8. IGHV gene repertoire of the tFL series.

IGHV gene	Frequency, n	Frequency, %	IGHV gene	Frequency, n	Frequency, %	IGHV gene	Frequency, n	Frequency, %
**IGHV1-2**	0	0,0	**IGHV3-30**	4	12,9	**IGHV4-38-2**	0	0,0
**IGHV1-3**	0	0,0	**IGHV3-30-3**	0	0,0	**IGHV4-39**	1	3,2
**IGHV1-8**	0	0,0	**IGHV3-30-5**	0	0,0	**IGHV4-55**	0	0,0
**IGHV1-18**	0	0,0	**IGHV3-33**	0	0,0	**IGHV4-59**	0	0,0
**IGHV1-24**	0	0,0	**IGHV3-43**	0	0,0	**IGHV4-61**	0	0,0
**IGHV1-45**	0	0,0	**IGHV3-43D**	0	0,0	**IGHV5-10-1**	0	0,0
**IGHV1-46**	0	0,0	**IGHV3-48**	6	19,4	**IGHV5-51**	1	3,2
**IGHV1-58**	0	0,0	**IGHV3-49**	0	0,0	**IGHV6-1**	0	0,0
**IGHV1-69**	1	3,2	**IGHV3-53**	0	0,0	**IGHV7-4-1**	0	0,0
**IGHV1-69-2**	0	0,0	**IGHV3-64**	0	0,0	**Totals**	**31**	**100,0**
**IGHV1-69D**	0	0,0	**IGHV3-64D**	0	0,0			
**IGHV2-5**	0	0,0	**IGHV3-66**	0	0,0			
**IGHV2-26**	0	0,0	**IGHV3-72**	0	0,0			
**IGHV2-70**	0	0,0	**IGHV3-73**	1	3,2			
**IGHV2-70D**	0	0,0	**IGHV3-74**	1	3,2			
**IGHV3-7**	1	3,2	**IGHV3-NL1**	0	0,0			
**IGHV3-9**	0	0,0	**IGHV4-4**	0	0,0			
**IGHV3-11**	0	0,0	**IGHV4-28**	0	0,0			
**IGHV3-13**	1	3,2	**IGHV4-30**	0	0,0			
**IGHV3-15**	1	3,2	**IGHV4-30-1**	0	0,0			
**IGHV3-20**	0	0,0	**IGHV4-30-2**	0	0,0			
**IGHV3-21**	4	12,9	**IGHV4-30-4**	0	0,0			
**IGHV3-23**	4	12,9	**IGHV4-31**	0	0,0			
**IGHV3-23D**	0	0,0	**IGHV4-34**	5	16,1			

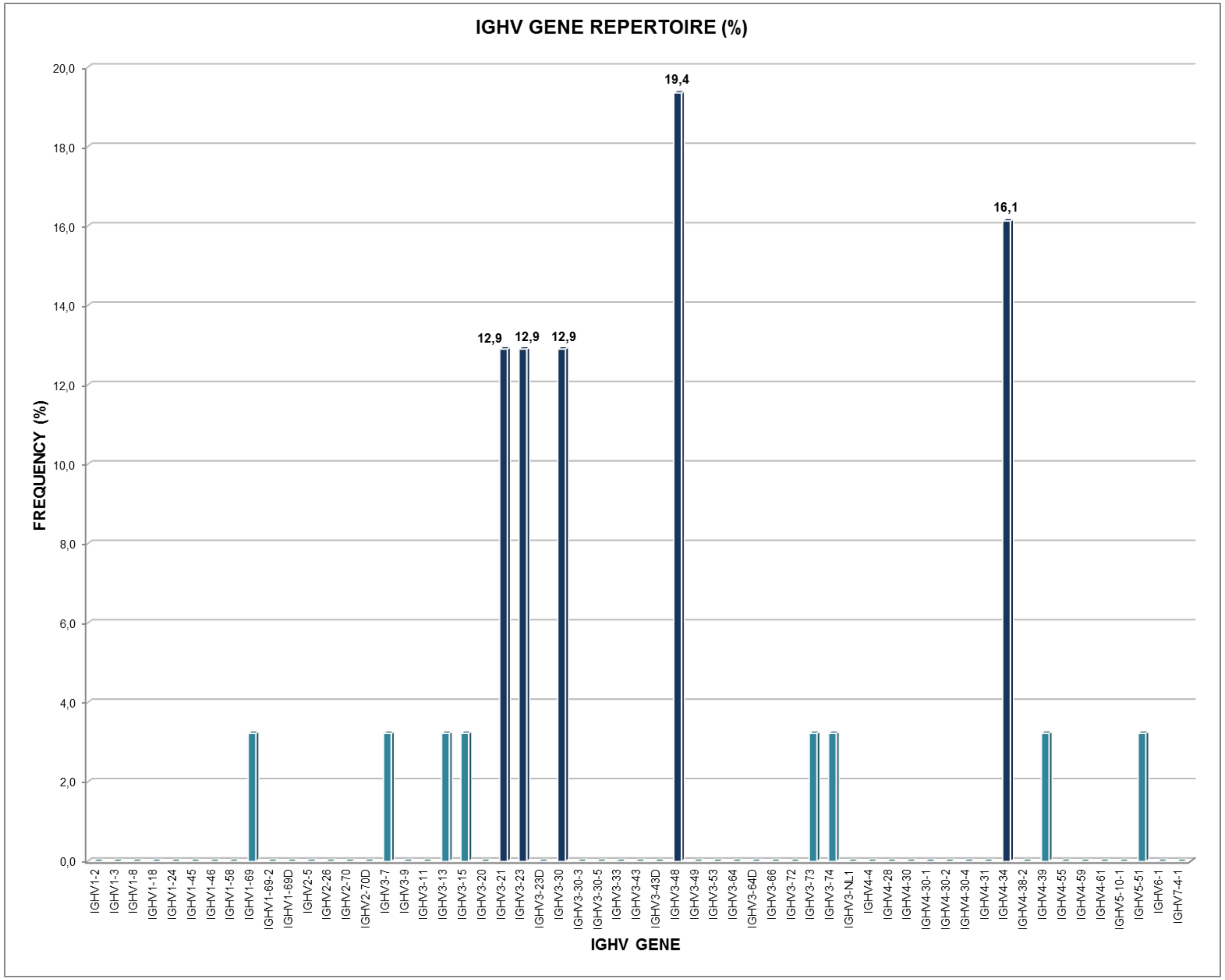
IGHV GENE REPERTOIRE (%)
FREQUENCY (%)
IGHV GENE
19,4
16,1
12,9
12,9
12,9
20,0
18,0
16,0
14,0
12,0
10,0
8,0
6,0
4,0
2,0
0,0
IGHV1-2 IGHV1-3 IGHV1-8 IGHV1-18 IGHV1-24 IGHV1-45 IGHV1-46 IGHV1-58 IGHV1-69 IGHV1-69-2 IGHV1-69D IGHV2-5 IGHV2-26 IGHV2-70 IGHV2-70D IGHV3-7 IGHV3-9 IGHV3-11 IGHV3-13 IGHV3-15 IGHV3-20 IGHV3-21 IGHV3-23 IGHV3-23D IGHV3-30 IGHV3-30-3 IGHV3-30-5 IGHV3-33 IGHV3-43 IGHV3-43D IGHV3-48 IGHV3-49 IGHV3-53 IGHV3-64 IGHV3-64D IGHV3-66 IGHV3-72 IGHV3-73 IGHV3-74 IGHV3-NL1 IGHV4-4 IGHV4-28 IGHV4-30 IGHV4-30-1 IGHV4-30-2 IGHV4-30-4 IGHV4-31 IGHV4-34 IGHV4-38-2 IGHV4-39 IGHV4-55 IGHV4-59 IGHV4-61 IGHV5-10-1 IGHV5-51 IGHV6-1 IGHV7-4-1

Table S9. IGHD gene repertoire of the tFL series.

IGHD gene	Frequency, n	Frequency, %	IGHD gene	Frequency, n	Frequency, %
**IGHD1-1**	0	0,0	**IGHD6-6**	1	4,0
**IGHD1-7**	0	0,0	**IGHD6-13**	1	4,0
**IGHD1-14**	0	0,0	**IGHD6-19**	0	0,0
**IGHD1-20**	1	4,0	**IGHD6-25**	2	8,0
**IGHD1-26**	2	8,0	**IGHD7-27**	0	0,0
**IGHD2-2**	3	12,0	**Totals**	**25**	**100,0**
**IGHD2-8**	0	0,0			
**IGHD2-15**	2	8,0			
**IGHD2-21**	3	12,0			
**IGHD3-3**	0	0,0			
**IGHD3-9**	1	4,0			
**IGHD3-10**	3	12,0			
**IGHD3-16**	1	4,0			
**IGHD3-22**	3	12,0			
**IGHD4-4**	0	0,0			
**IGHD4-11**	1	4,0			
**IGHD4-17**	0	0,0			
**IGHD4-23**	1	4,0			
**IGHD5-5**	0	0,0			
**IGHD5-12**	0	0,0			
**IGHD5-18**	0	0,0			
**IGHD5-24**	0	0,0			

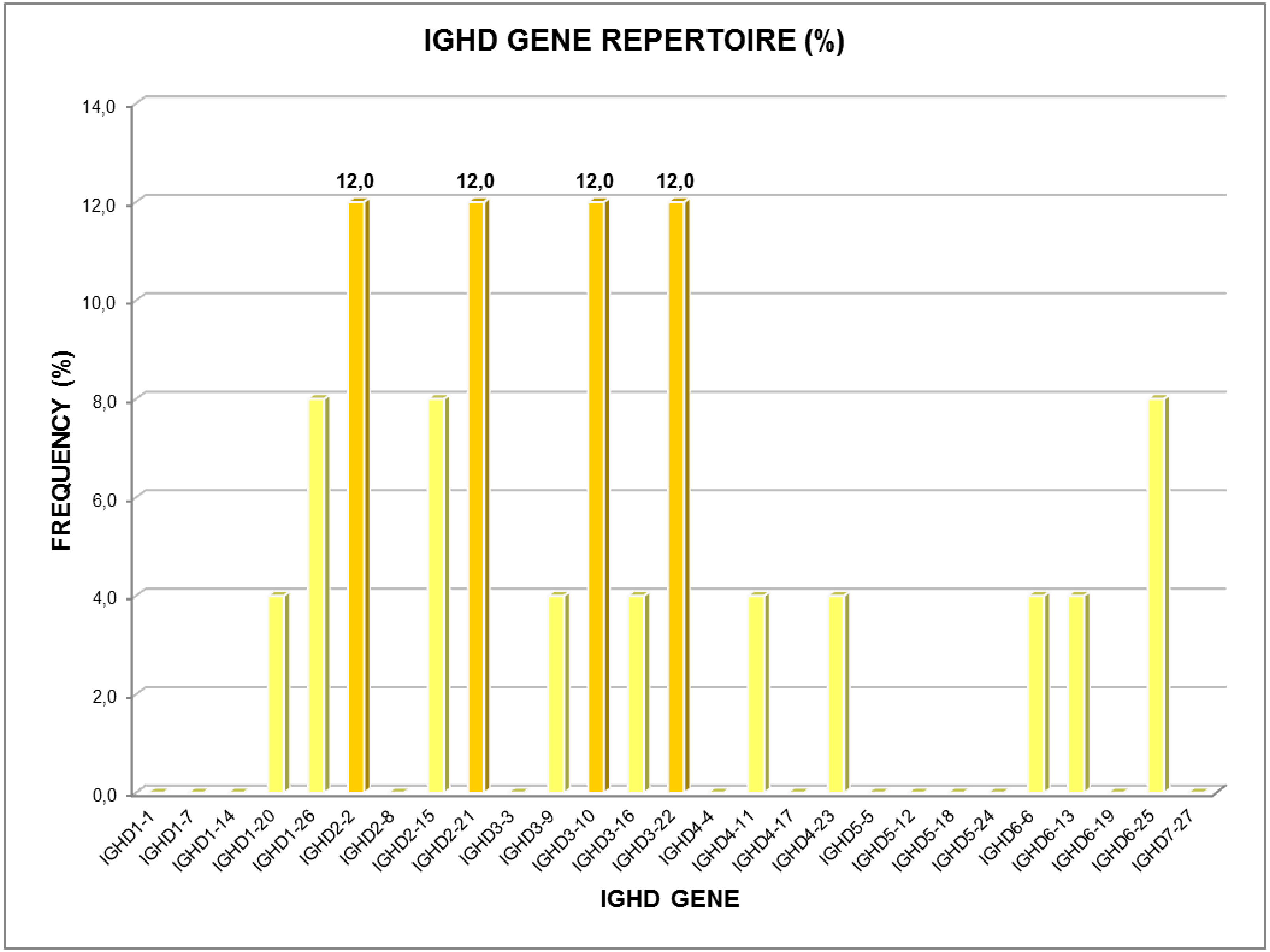
IGHD GENE REPERTOIRE (%)
FREQUENCY (%)
IGHD GENE
14,0
12,0
10,0
8,0
6,0
4,0
2,0
0,0
12,0
12,0
12,0
12,0
IGHD1-1
IGHD1-7
IGHD1-14
IGHD1-20
IGHD1-26
IGHD2-2
IGHD2-8
IGHD2-15
IGHD2-21
IGHD3-3
IGHD3-9
IGHD3-10
IGHD3-16
IGHD3-22
IGHD4-4
IGHD4-11
IGHD4-17
IGHD4-23
IGHD5-5
IGHD5-12
IGHD5-18
IGHD5-24
IGHD6-6
IGHD6-13
IGHD6-19
IGHD6-25
IGHD7-27

Table S10. Comparative analysis of the IGHV usage in tFL with FL and GCB/non-GCB DLBCL.

IGH	tFL (n=31)		FL (n=63)		*P*	GCB DLBCL (n=32)[1]		*P*	non-GCB DLBCL (n=48)[1]		*P*
	n	%	n	%		n	%		n	%	
**IGHV**											
1	1	3,2	3	4,8	1,0000	4	12,5	0,3540	11	22,9	0,0245
2	0	0,0	2	3,2	1,0000	1	3,1	0,4918	1	2,1	1,0000
3	23	74,2	40	63,5	0,3560	19	59,4	0,2831	19	39,6	0,0027
4	6	19,4	17	27,0	0,4580	6	18,8	1,0000	15	31,3	0,1373
5	1	3,2	0	0,0	0,3298	1	3,1	1,0000	2	4,2	1,0000
6	0	0,0	1	1,6	1,0000	1	3,1	0,4918	0	0,0	1,0000
7	0	0,0	0	0,0	1,0000	0	0,0	1,0000	0	0,0	1,0000
3-21	4	12,9	5	7,9	0,4710	2	6,3	0,6713	1	2,1	0,0680
3-23	4	12,9	8	12,7	1,0000	3	9,4	1,0000	6	12,5	1,0000
3-30	4	12,9	9	14,3	1,0000	0	0,0	0,1128	0	0,0	0,0189
3-48	6	19,4	3	4,8	0,0551	2	6,3	0,2554	2	4,2	0,0491
4-34	5	16,1	8	12,7	0,7528	0	0,0	0,0525	12	25,0	0,2853

References: **1**-Diffuse Large B-Cell Lymphoma, Sebastian et al, American Journal of Pathology 2012

Table S11. Comparison of the IGHV gene usage in tFL with FL and GCB/non-GCB DLBCL.

tFL			FL			GCB DLBCL[1]			non-GCB DLBCL[1]		
IGHV gene	Frequency, n	Frequency, %	IGHV gene	Frequency, n	Frequency, %	IGHV gene	Frequency, n	Frequency, %	IGHV gene	Frequency, n	Frequency, %
**IGHV1-2**	0	0,0	**IGHV1-2**	0	0,0	**IGHV1-2**	0	0,0	**IGHV1-2**	3	6,3
**IGHV1-3**	0	0,0	**IGHV1-3**	0	0,0	**IGHV1-3**	0	0,0	**IGHV1-3**	1	2,1
**IGHV1-8**	0	0,0	**IGHV1-8**	1	1,6	**IGHV1-8**	1	3,1	**IGHV1-8**	1	2,1
**IGHV1-18**	0	0,0	**IGHV1-18**	0	0,0	**IGHV1-18**	1	3,1	**IGHV1-18**	2	4,2
**IGHV1-24**	0	0,0	**IGHV1-24**	0	0,0	**IGHV1-24**	0	0,0	**IGHV1-24**	0	0,0
**IGHV1-45**	0	0,0	**IGHV1-45**	0	0,0	**IGHV1-45**	0	0,0	**IGHV1-45**	0	0,0
**IGHV1-46**	0	0,0	**IGHV1-46**	1	1,6	**IGHV1-46**	1	3,1	**IGHV1-46**	1	2,1
**IGHV1-58**	0	0,0	**IGHV1-58**	0	0,0	**IGHV1-58**	0	0,0	**IGHV1-58**	0	0,0
**IGHV1-69**	1	3,2	**IGHV1-69**	1	1,6	**IGHV1-69**	1	3,1	**IGHV1-69**	3	6,3
**IGHV1-69-2**	0	0,0	**IGHV1-69-2**	0	0,0	**IGHV1-69-2**	0	0,0	**IGHV1-69-2**	0	0,0
**IGHV1-69D**	0	0,0	**IGHV1-69D**	0	0,0	**IGHV1-69D**	0	0,0	**IGHV1-69D**	0	0,0
**IGHV2-5**	0	0,0	**IGHV2-5**	1	1,6	**IGHV2-5**	1	3,1	**IGHV2-5**	1	2,1
**IGHV2-26**	0	0,0	**IGHV2-26**	0	0,0	**IGHV2-26**	0	0,0	**IGHV2-26**	0	0,0
**IGHV2-70**	0	0,0	**IGHV2-70**	1	1,6	**IGHV2-70**	0	0,0	**IGHV2-70**	0	0,0
**IGHV2-70D**	0	0,0	**IGHV2-70D**	0	0,0	**IGHV2-70D**	0	0,0	**IGHV2-70D**	0	0,0
**IGHV3-7**	1	3,2	**IGHV3-7**	4	6,3	**IGHV3-7**	2	6,3	**IGHV3-7**	2	4,2
**IGHV3-9**	0	0,0	**IGHV3-9**	0	0,0	**IGHV3-9**	0	0,0	**IGHV3-9**	2	4,2
**IGHV3-11**	0	0,0	**IGHV3-11**	3	4,8	**IGHV3-11**	2	6,3	**IGHV3-11**	0	0,0
**IGHV3-13**	1	3,2	**IGHV3-13**	0	0,0	**IGHV3-13**	1	3,1	**IGHV3-13**	0	0,0
**IGHV3-15**	1	3,2	**IGHV3-15**	2	3,2	**IGHV3-15**	0	0,0	**IGHV3-15**	1	2,1
**IGHV3-20**	0	0,0	**IGHV3-20**	1	1,6	**IGHV3-20**	1	3,1	**IGHV3-20**	0	0,0
**IGHV3-21**	4	12,9	**IGHV3-21**	5	7,9	**IGHV3-21**	2	6,3	**IGHV3-21**	1	2,1

**IGHV3-23**	4	12,9	**IGHV3-23**	8	12,7	**IGHV3-23**	3	9,4	**IGHV3-23**	6	12,5
**IGHV3-23D**	0	0,0	**IGHV3-23D**	0	0,0	**IGHV3-23D**	0	0,0	**IGHV3-23D**	0	0,0
**IGHV3-30**	4	12,9	**IGHV3-30**	9	14,3	**IGHV3-30**	0	0,0	**IGHV3-30**	0	0,0
**IGHV3-30-3**	0	0,0	**IGHV3-30-3**	0	0,0	**IGHV3-30-3**	0	0,0	**IGHV3-30-3**	0	0,0
**IGHV3-30-5**	0	0,0	**IGHV3-30-5**	0	0,0	**IGHV3-30-5**	0	0,0	**IGHV3-30-5**	0	0,0
**IGHV3-33**	0	0,0	**IGHV3-33**	4	6,3	**IGHV3-33**	3	9,4	**IGHV3-33**	0	0,0
**IGHV3-43**	0	0,0	**IGHV3-43**	0	0,0	**IGHV3-43**	0	0,0	**IGHV3-43**	0	0,0
**IGHV3-43D**	0	0,0	**IGHV3-43D**	0	0,0	**IGHV3-43D**	0	0,0	**IGHV3-43D**	0	0,0
**IGHV3-48**	6	19,4	**IGHV3-48**	3	4,8	**IGHV3-48**	2	6,3	**IGHV3-48**	2	4,2
**IGHV3-49**	0	0,0	**IGHV3-49**	0	0,0	**IGHV3-49**	0	0,0	**IGHV3-49**	0	0,0
**IGHV3-53**	0	0,0	**IGHV3-53**	1	1,6	**IGHV3-53**	2	6,3	**IGHV3-53**	4	8,3
**IGHV3-64**	0	0,0	**IGHV3-64**	0	0,0	**IGHV3-64**	0	0,0	**IGHV3-64**	0	0,0
**IGHV3-64D**	0	0,0	**IGHV3-64D**	0	0,0	**IGHV3-64D**	0	0,0	**IGHV3-64D**	0	0,0
**IGHV3-66**	0	0,0	**IGHV3-66**	0	0,0	**IGHV3-66**	0	0,0	**IGHV3-66**	0	0,0
**IGHV3-72**	0	0,0	**IGHV3-72**	0	0,0	**IGHV3-72**	0	0,0	**IGHV3-72**	0	0,0
**IGHV3-73**	1	3,2	**IGHV3-73**	0	0,0	**IGHV3-73**	0	0,0	**IGHV3-73**	0	0,0
**IGHV3-74**	1	3,2	**IGHV3-74**	0	0,0	**IGHV3-74**	1	3,1	**IGHV3-74**	0	0,0
**IGHV3-NL1**	0	0,0	**IGHV3-NL1**	0	0,0	**IGHV3-NL1**	0	0,0	**IGHV3-NL1**	0	0,0
**IGHV4-4**	0	0,0	**IGHV4-4**	0	0,0	**IGHV4-4**	0	0,0	**IGHV4-4**	0	0,0
**IGHV4-28**	0	0,0	**IGHV4-28**	0	0,0	**IGHV4-28**	1	3,1	**IGHV4-28**	0	0,0
**IGHV4-30**	0	0,0	**IGHV4-30**	1	1,6	**IGHV4-30**	0	0,0	**IGHV4-30**	0	0,0
**IGHV4-30-1**	0	0,0	**IGHV4-30-1**	0	0,0	**IGHV4-30-1**	0	0,0	**IGHV4-30-1**	0	0,0
**IGHV4-30-2**	0	0,0	**IGHV4-30-2**	0	0,0	**IGHV4-30-2**	0	0,0	**IGHV4-30-2**	0	0,0
**IGHV4-30-4**	0	0,0	**IGHV4-30-4**	0	0,0	**IGHV4-30-4**	0	0,0	**IGHV4-30-4**	0	0,0
**IGHV4-31**	0	0,0	**IGHV4-31**	0	0,0	**IGHV4-31**	0	0,0	**IGHV4-31**	0	0,0
**IGHV4-34**	5	16,1	**IGHV4-34**	8	12,7	**IGHV4-34**	0	0,0	**IGHV4-34**	12	25,0
**IGHV4-38-2**	0	0,0	**IGHV4-38-2**	0	0,0	**IGHV4-38-2**	0	0,0	**IGHV4-38-2**	0	0,0

**IGHV4-39**	1	3,2	**IGHV4-39**	3	4,8	**IGHV4-39**	3	9,4	**IGHV4-39**	2	4,2
**IGHV4-55**	0	0,0	**IGHV4-55**	1	1,6	**IGHV4-55**	0	0,0	**IGHV4-55**	0	0,0
**IGHV4-59**	0	0,0	**IGHV4-59**	1	1,6	**IGHV4-59**	1	3,1	**IGHV4-59**	1	2,1
**IGHV4-61**	0	0,0	**IGHV4-61**	3	4,8	**IGHV4-61**	1	3,1	**IGHV4-61**	1	2,1
**IGHV5-1**	0	0,0	**IGHV5-1**	0	0,0	**IGHV5-1**	0	0,0	**IGHV5-1**	1	2,1
**IGHV5-10-1**	0	0,0	**IGHV5-10-1**	0	0,0	**IGHV5-10-1**	0	0,0	**IGHV5-10-1**	0	0,0
**IGHV5-51**	1	3,2	**IGHV5-51**	0	0,0	**IGHV5-51**	1	3,1	**IGHV5-51**	1	2,1
**IGHV6-1**	0	0,0	**IGHV6-1**	1	1,6	**IGHV6-1**	1	3,1	**IGHV6-1**	0	0,0
**IGHV7-4-1**	0	0,0	**IGHV7-4-1**	0	0,0	**IGHV7-4-1**	0	0,0	**IGHV7-4-1**	0	0,0
**Totals**	**31**	**100,0**	**Totals**	**63**	**100,0**	**Totals**	**32**	**100,0**	**Totals**	**48**	**100,0**

References: 1-Diffuse Large B-Cell Lymphoma, Sebastian et al, American Journal of Pathology 2012

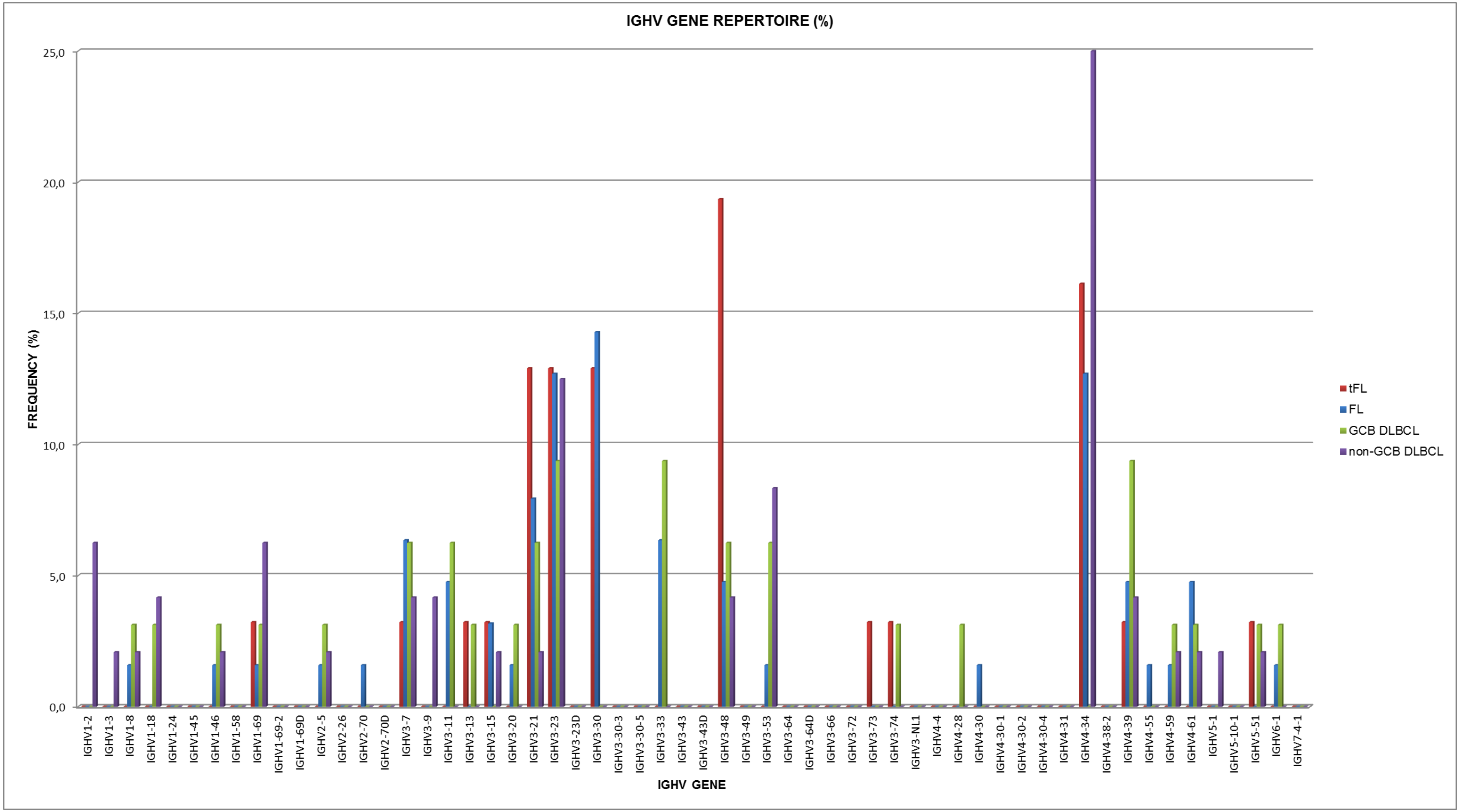
IGHV GENE REPERTOIRE (%)
FREQUENCY (%)
25,0
20,0
15,0
10,0
5,0
0,0
IGHV1-2
IGHV1-3
IGHV1-8
IGHV1-18
IGHV1-24
IGHV1-45
IGHV1-46
IGHV1-58
IGHV1-69
IGHV1-69-2
IGHV1-69D
IGHV2-5
IGHV2-26
IGHV2-70
IGHV2-70D
IGHV3-7
IGHV3-9
IGHV3-11
IGHV3-13
IGHV3-15
IGHV3-20
IGHV3-21
IGHV3-23
IGHV3-23D
IGHV3-30
IGHV3-30-3
IGHV3-30-5
IGHV3-33
IGHV3-43
IGHV3-43D
IGHV3-48
IGHV3-49
IGHV3-53
IGHV3-64
IGHV3-64D
IGHV3-66
IGHV3-72
IGHV3-73
IGHV3-74
IGHV3-NL1
IGHV4-4
IGHV4-28
IGHV4-30
IGHV4-30-1
IGHV4-30-2
IGHV4-30-4
IGHV4-31
IGHV4-34
IGHV4-38-2
IGHV4-39
IGHV4-55
IGHV4-59
IGHV4-61
IGHV5-1
IGHV5-10-1
IGHV5-51
IGHV6-1
IGHV7-4-1
IGHV GENE
tFL
FL
GCB DLBCL
non-GCB DLBCL
